# Supplementary figures and images for: Cannabinoid combination targets NOTCH1-mutated T-cell acute lymphoblastic leukemia through the integrated stress response pathway
Source: eLife. 2024 Sep 11;12:RP90854. doi: 10.7554/eLife.90854 (PMC11390110; doi:10.7554/eLife.90854)

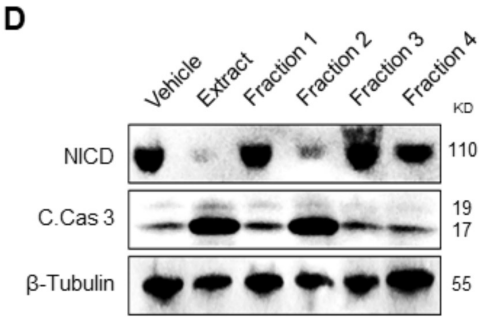

**NICD**

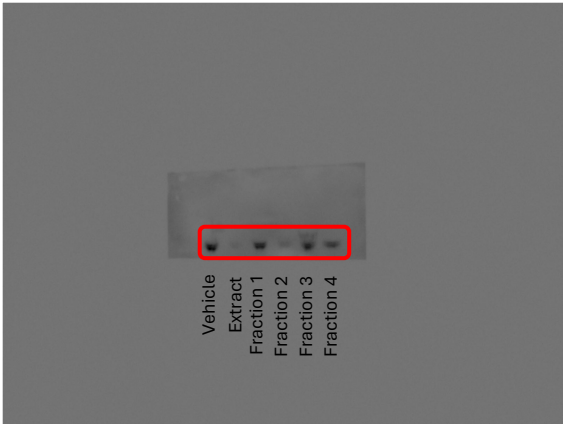

**C. Caspase 3**

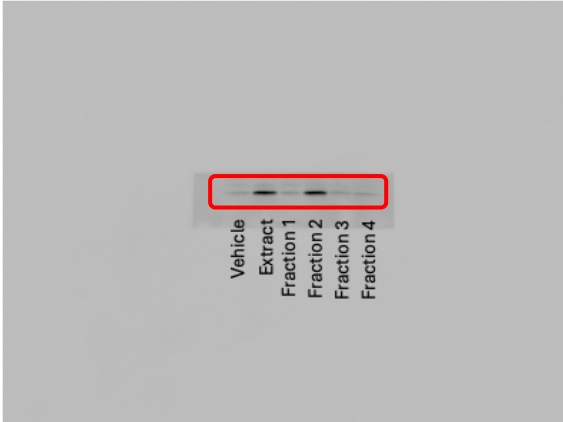

**Tubulin**

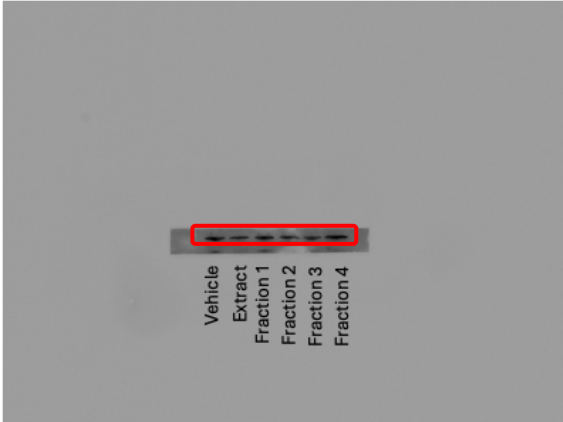

Supplement: Figure 1—source data 1. [file elife-90854-fig1-data1.zip › Fig 1 D/Figure 1 D.pdf]

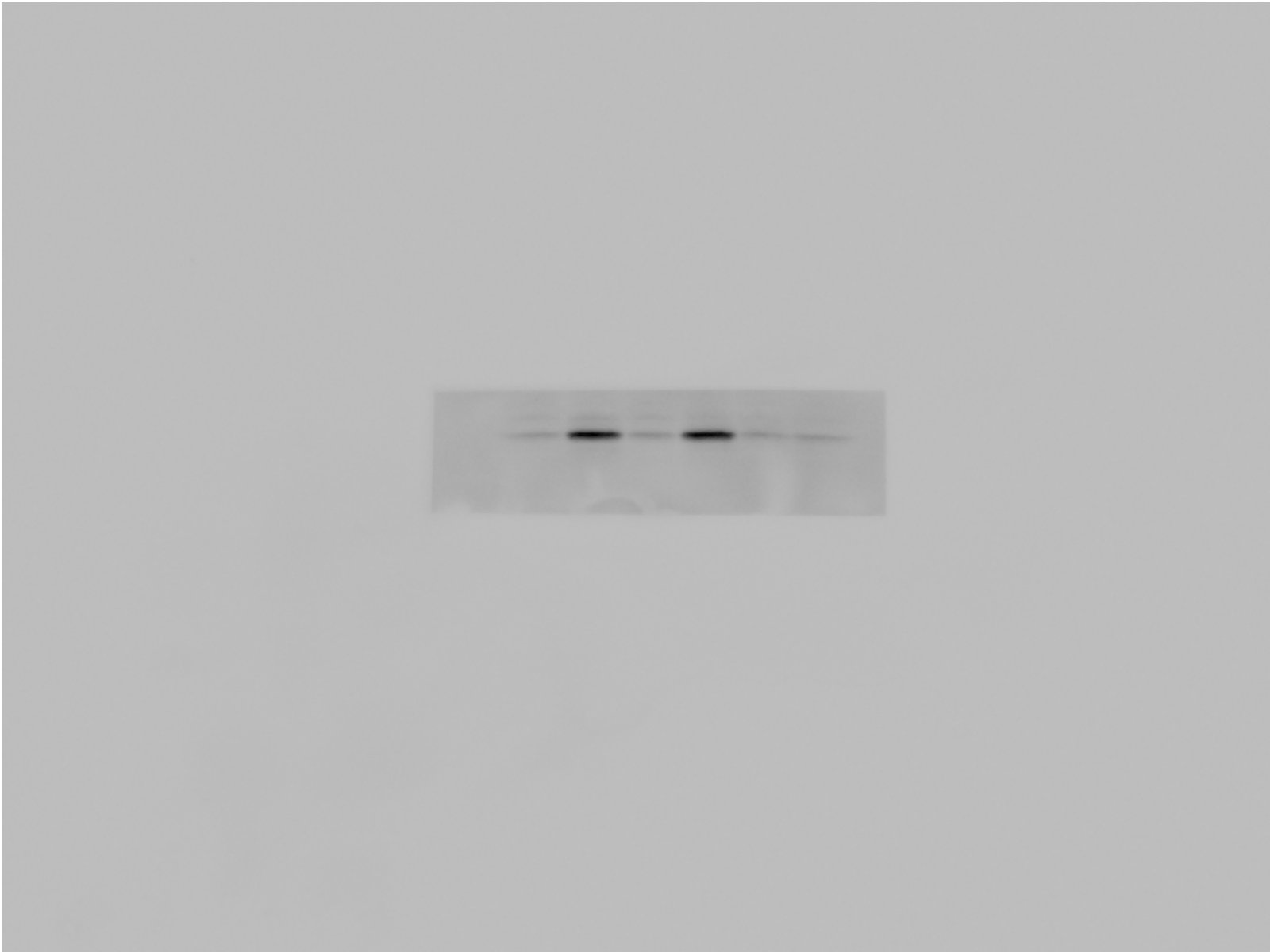

Supplement: Figure 1—source data 1. [file elife-90854-fig1-data1.zip › Fig 1 D/m casp3 inv.jpg]

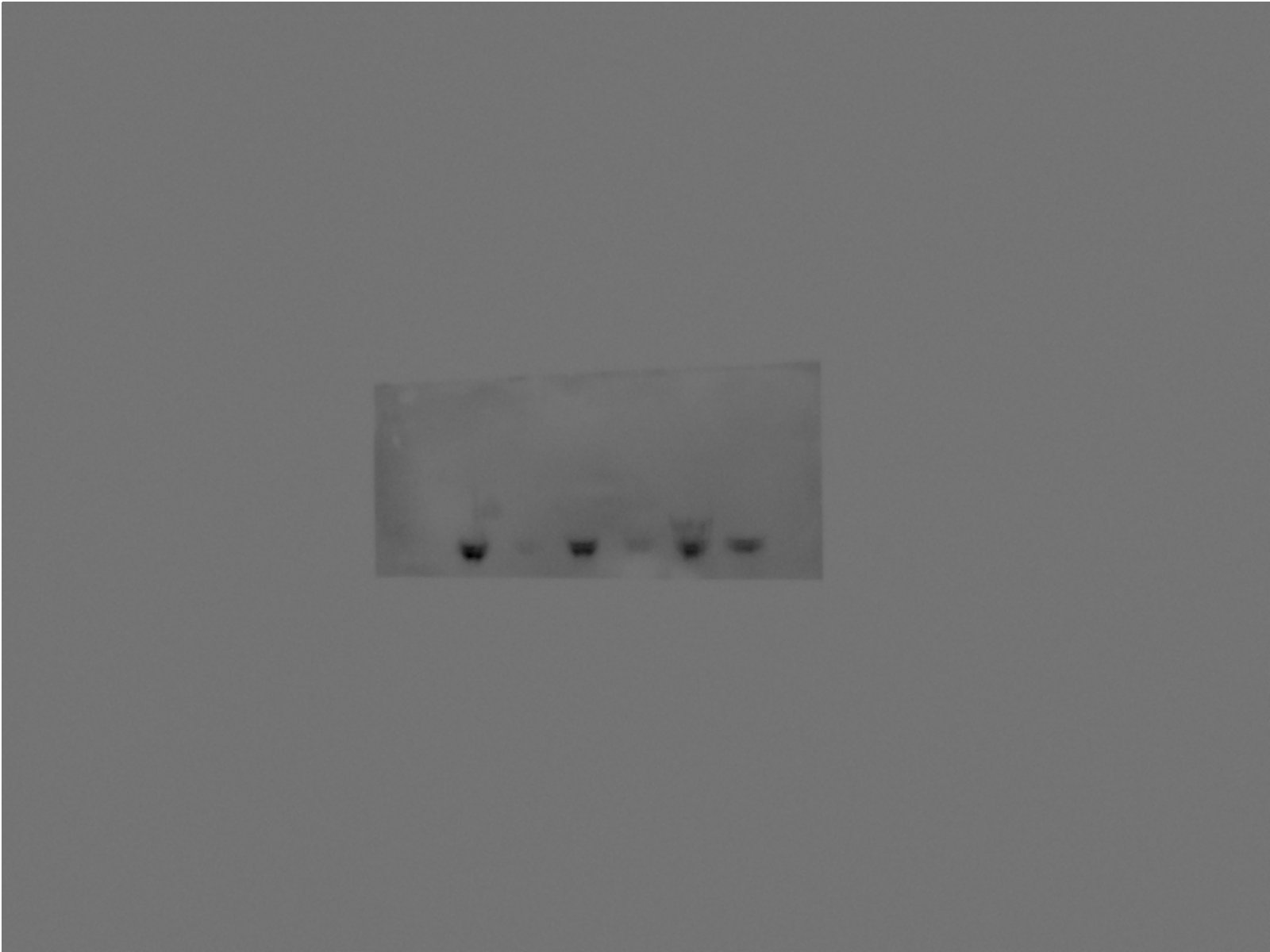

Supplement: Figure 1—source data 1. [file elife-90854-fig1-data1.zip › Fig 1 D/m nicd.jpg]

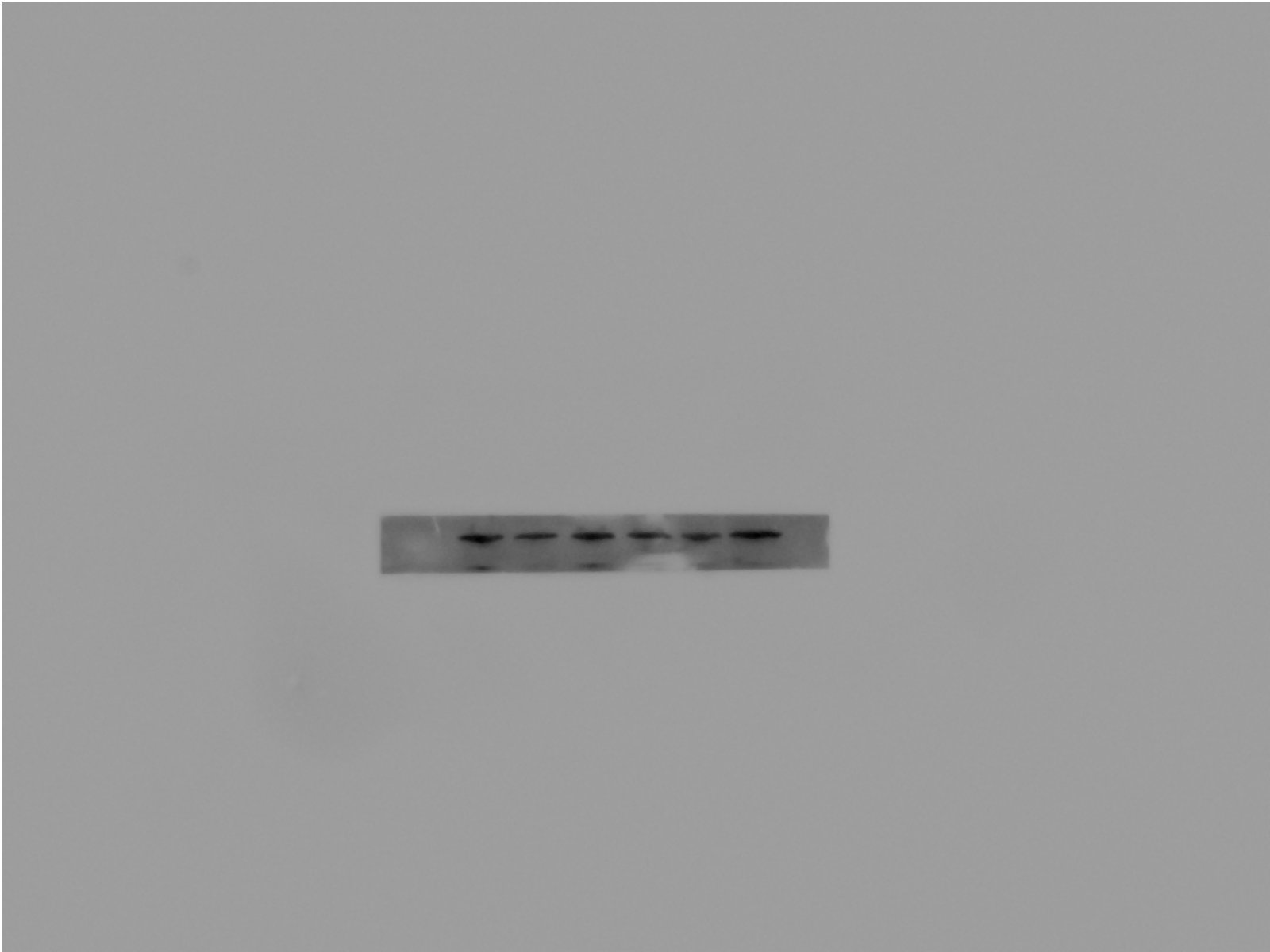

Supplement: Figure 1—source data 1. [file elife-90854-fig1-data1.zip › Fig 1 D/m Tubulin.jpg]

I

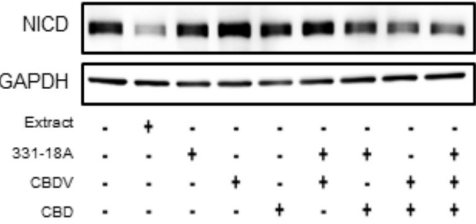

NICD

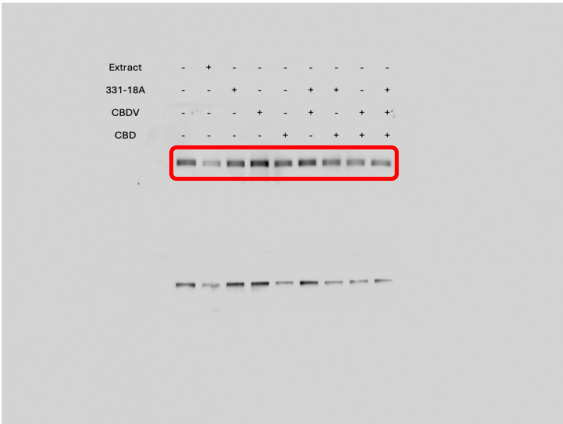

GAPDH

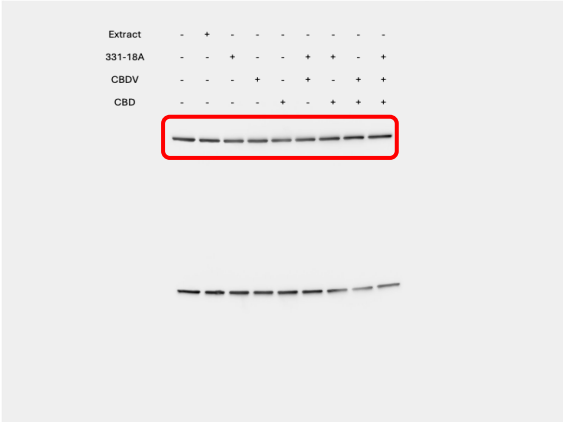

Supplement: Figure 1—source data 2. [file elife-90854-fig1-data2.zip › Fig 1 I/Figure 1 I.pdf]

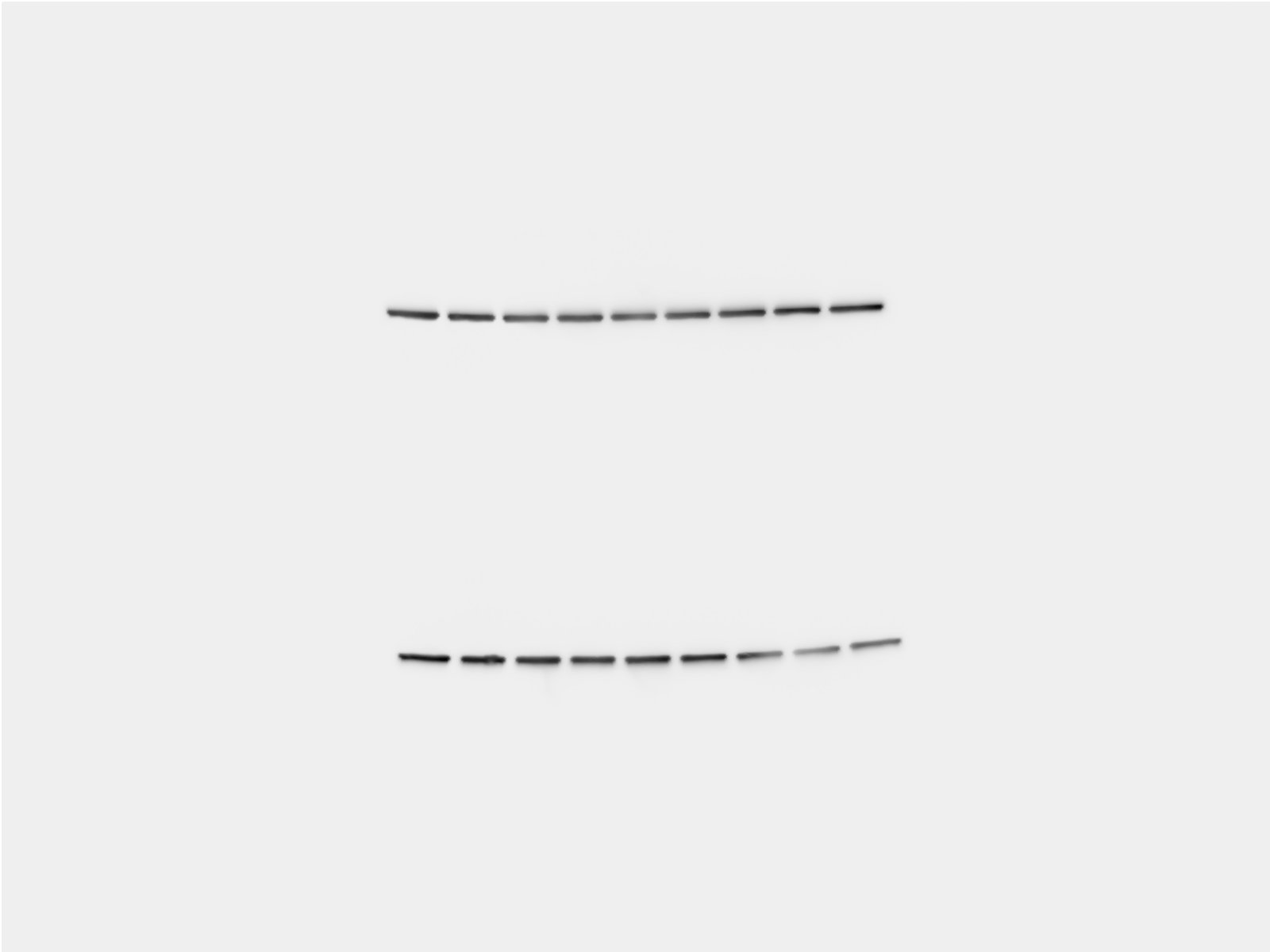

Supplement: Figure 1—source data 2. [file elife-90854-fig1-data2.zip › Fig 1 I/GAPDH 3.jpg]

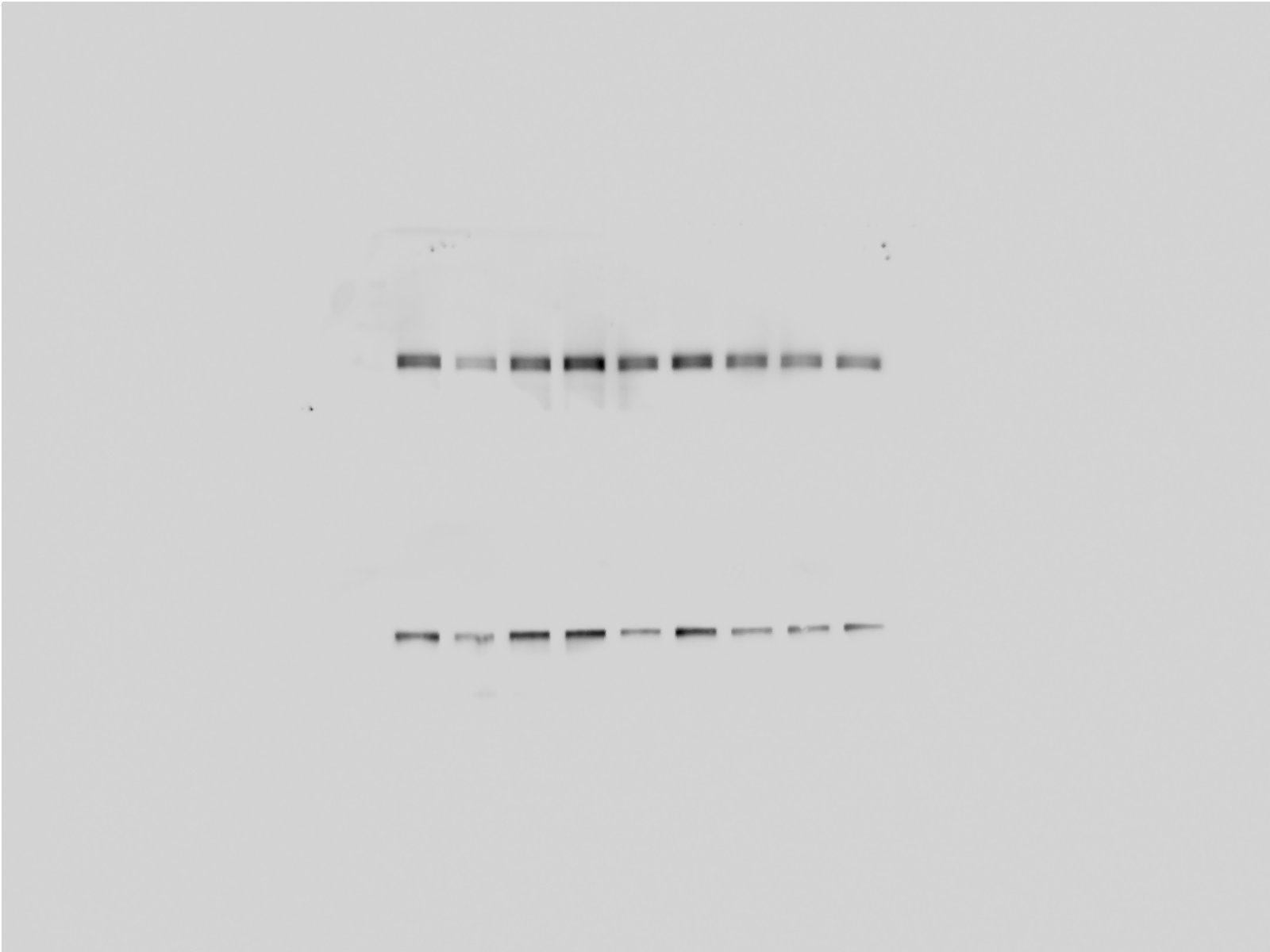

Supplement: Figure 1—source data 2. [file elife-90854-fig1-data2.zip › Fig 1 I/NICD 1.jpg]

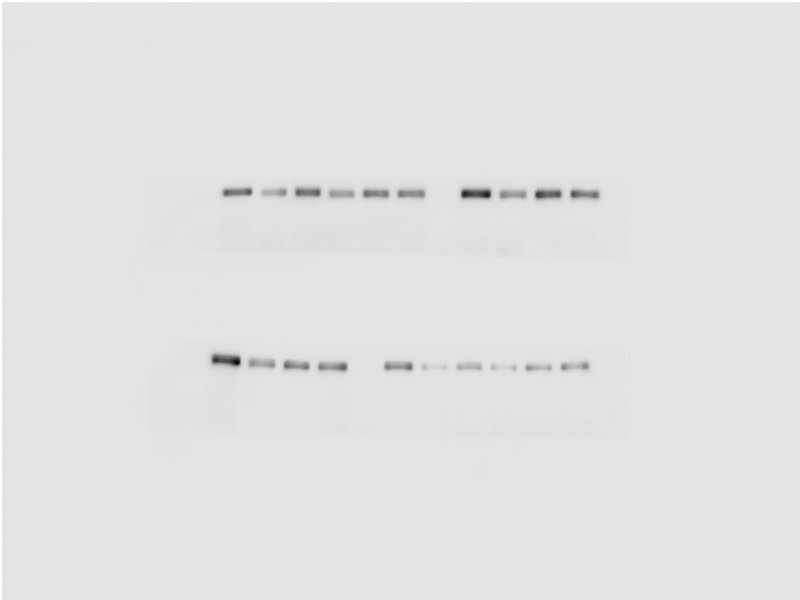

Supplement: Figure 2—source data 1. [file elife-90854-fig2-data1.zip › Fig 2 - source data 1/NICD_1 M 630_9810.jpg]

C

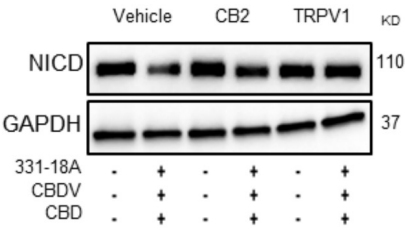

NICD

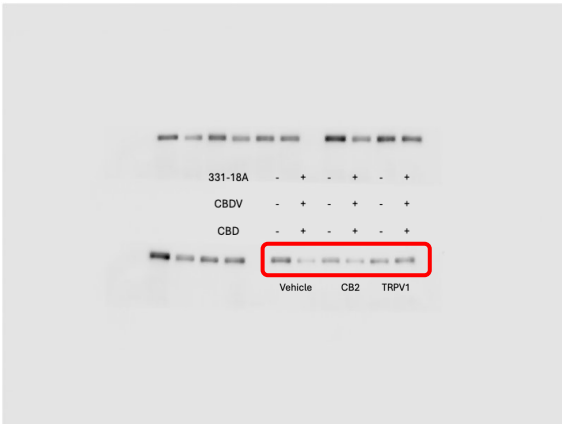

GAPDH

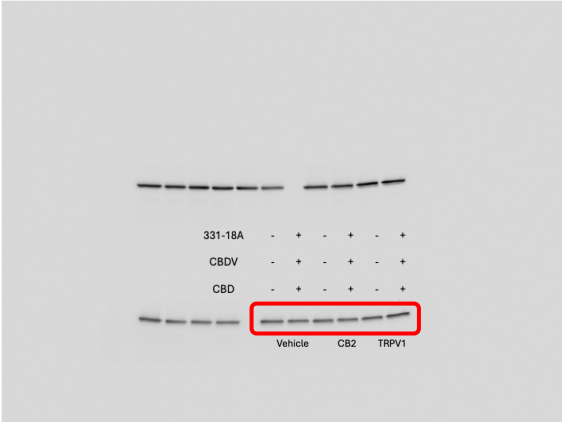

Supplement: Figure 2—source data 1. [file elife-90854-fig2-data1.zip › Fig 2 - source data 1/Figure 2 C 0624.pdf]

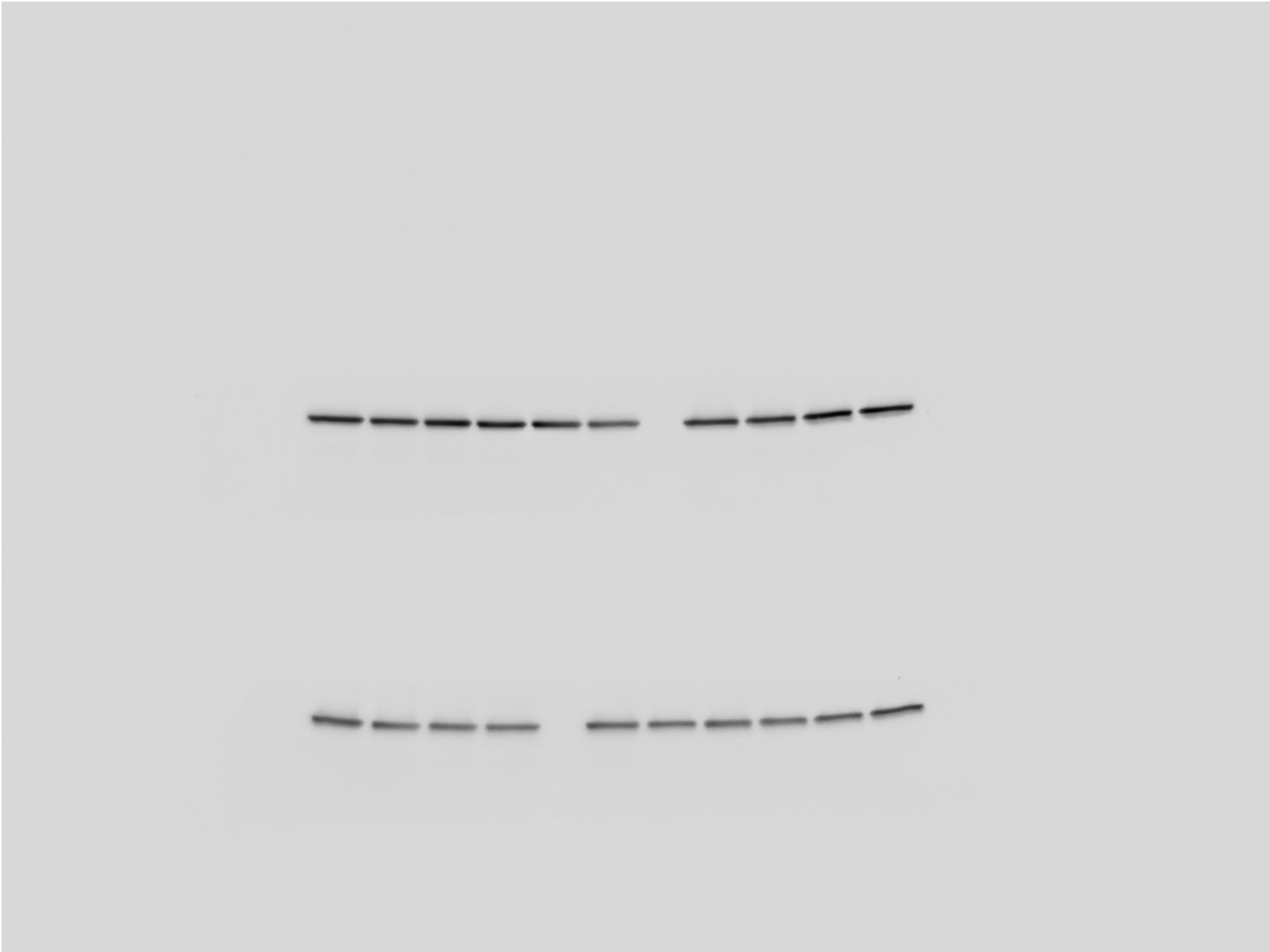

Supplement: Figure 2—source data 1. [file elife-90854-fig2-data1.zip › Fig 2 - source data 1/GAPDH M 630_9810.jpg]

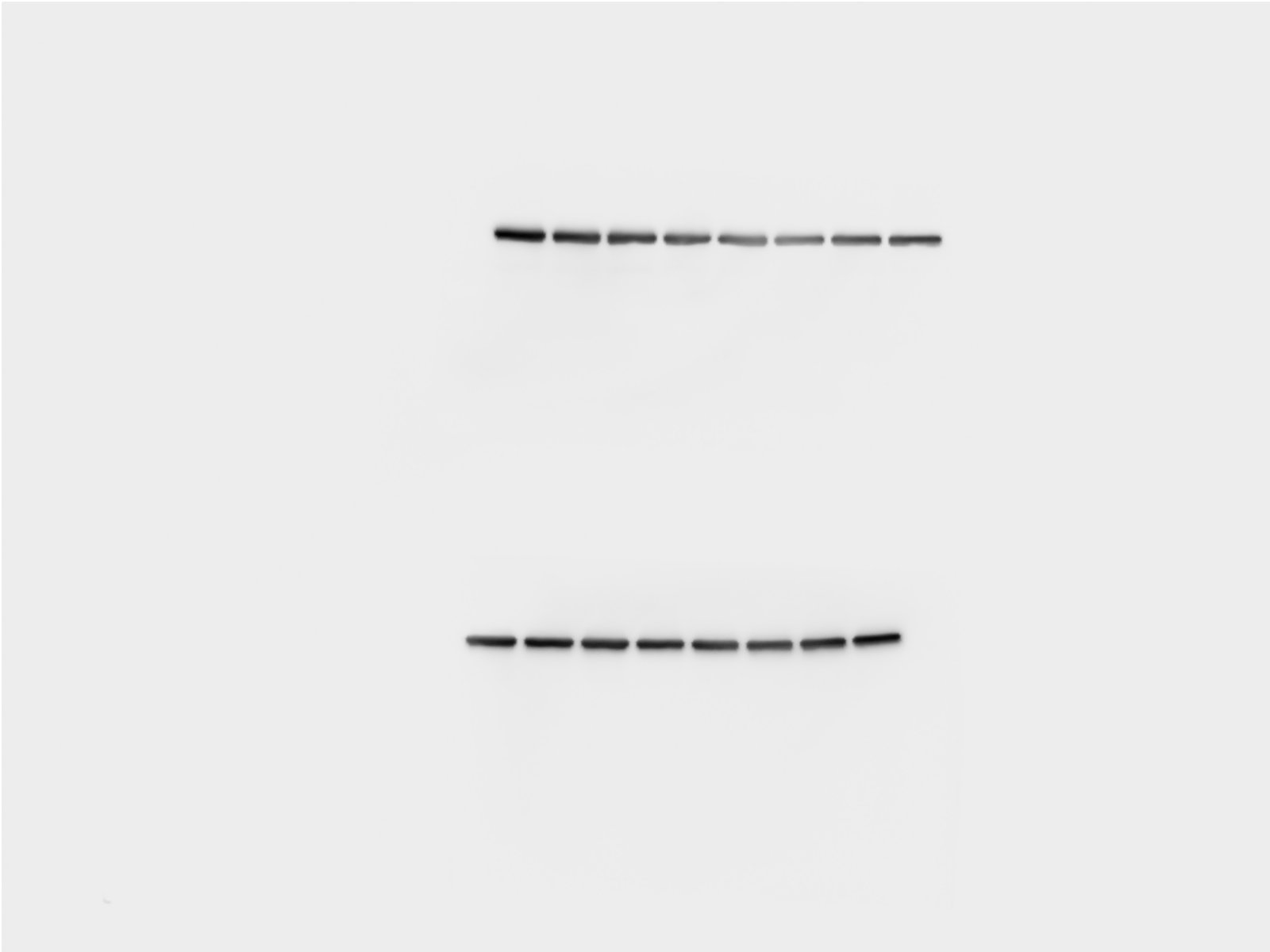

Supplement: Figure 2—figure supplement 1—source data 1. [file elife-90854-fig2-figsupp1-data1.zip › S4 B/BIM gapdh.jpg]

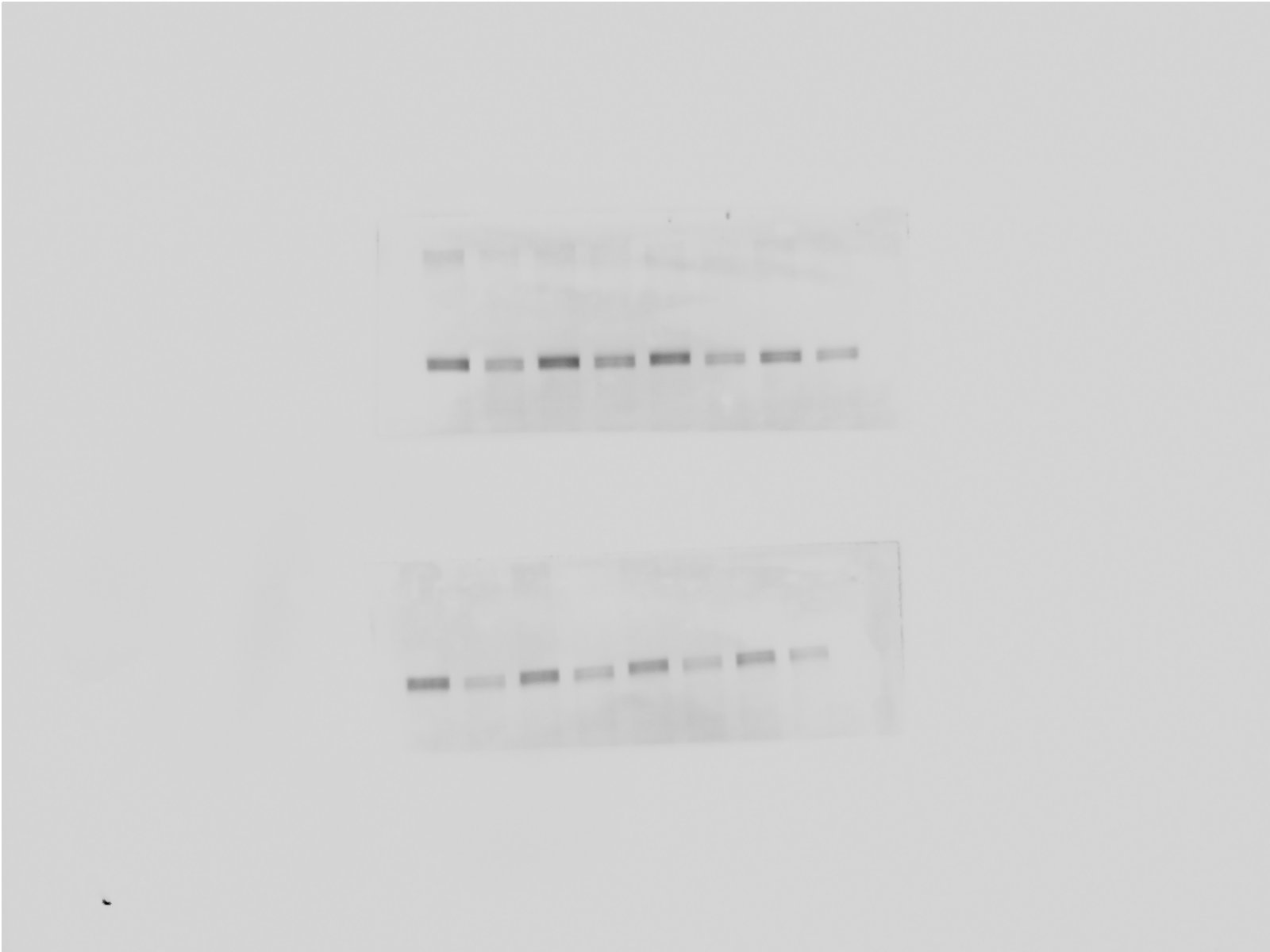

Supplement: Figure 2—figure supplement 1—source data 1. [file elife-90854-fig2-figsupp1-data1.zip › S4 B/BIM nicd 2.jpg]

NICD

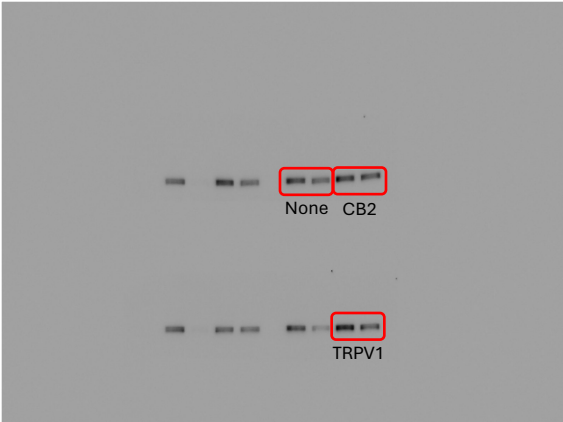

B

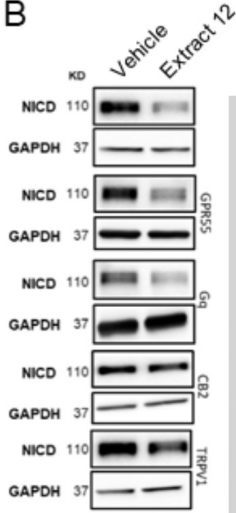

NICD

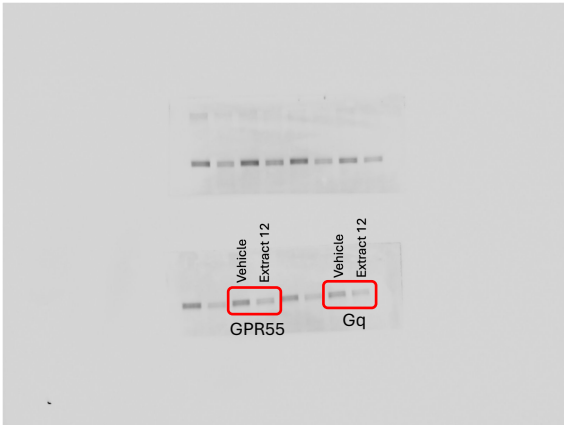

GAPDH

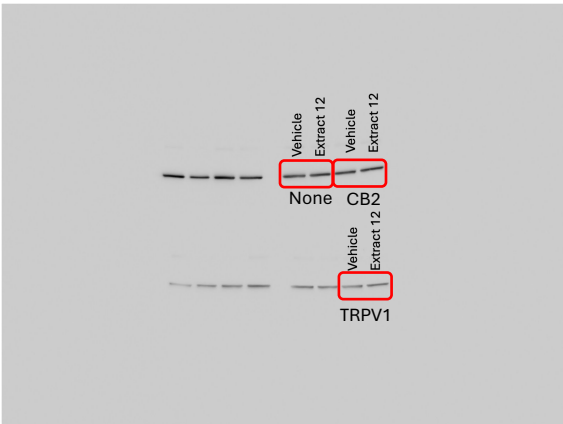

GAPDH

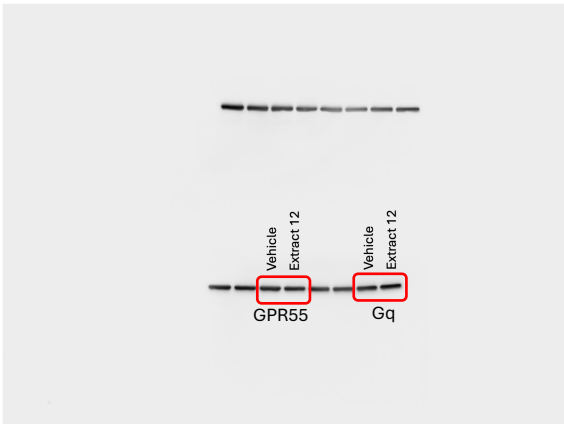

Supplement: Figure 2—figure supplement 1—source data 1. [file elife-90854-fig2-figsupp1-data1.zip › S4 B/Figure S4 B.pdf]

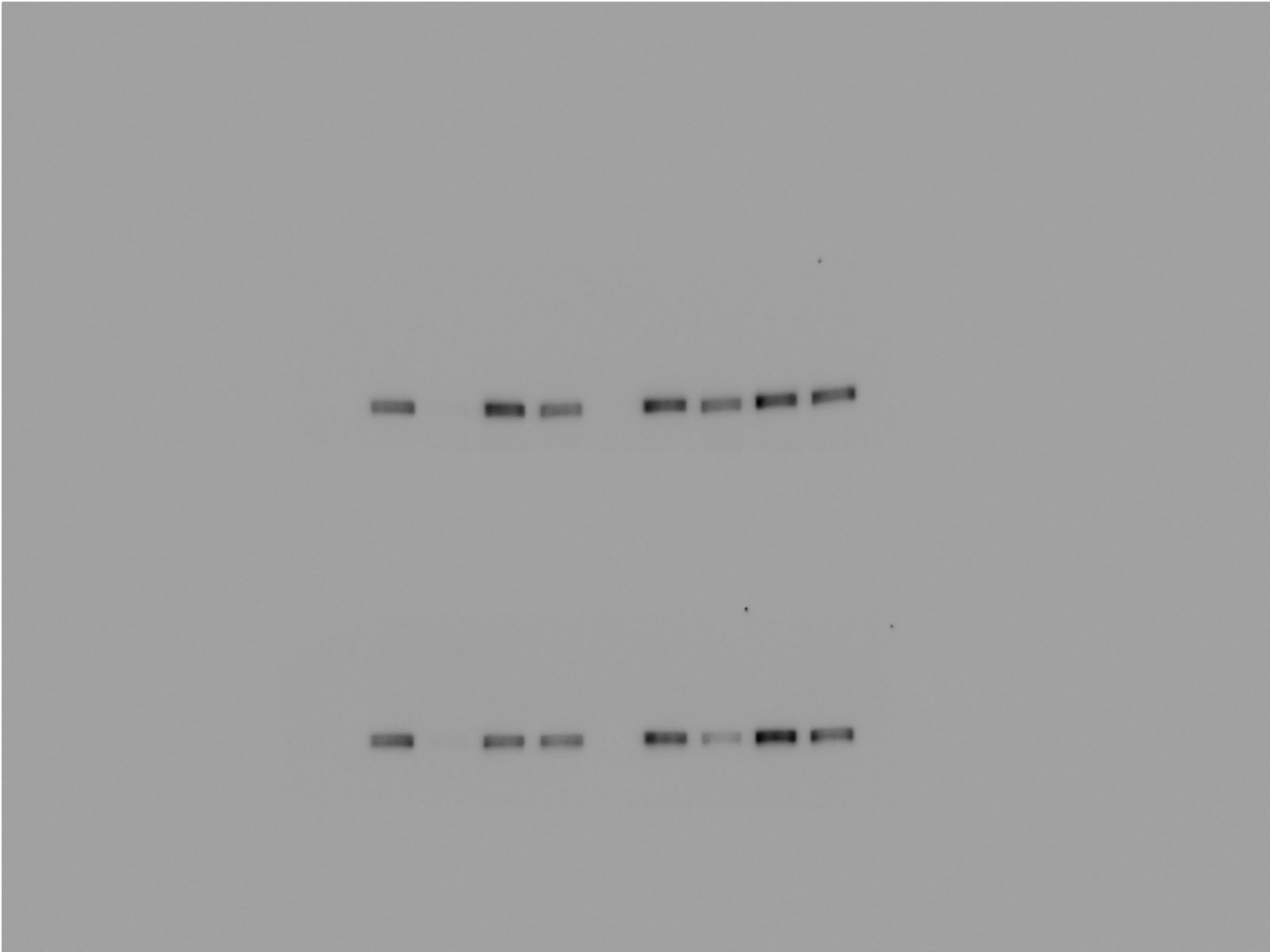

Supplement: Figure 2—figure supplement 1—source data 1. [file elife-90854-fig2-figsupp1-data1.zip › S4 B/NICD inv.jpg]

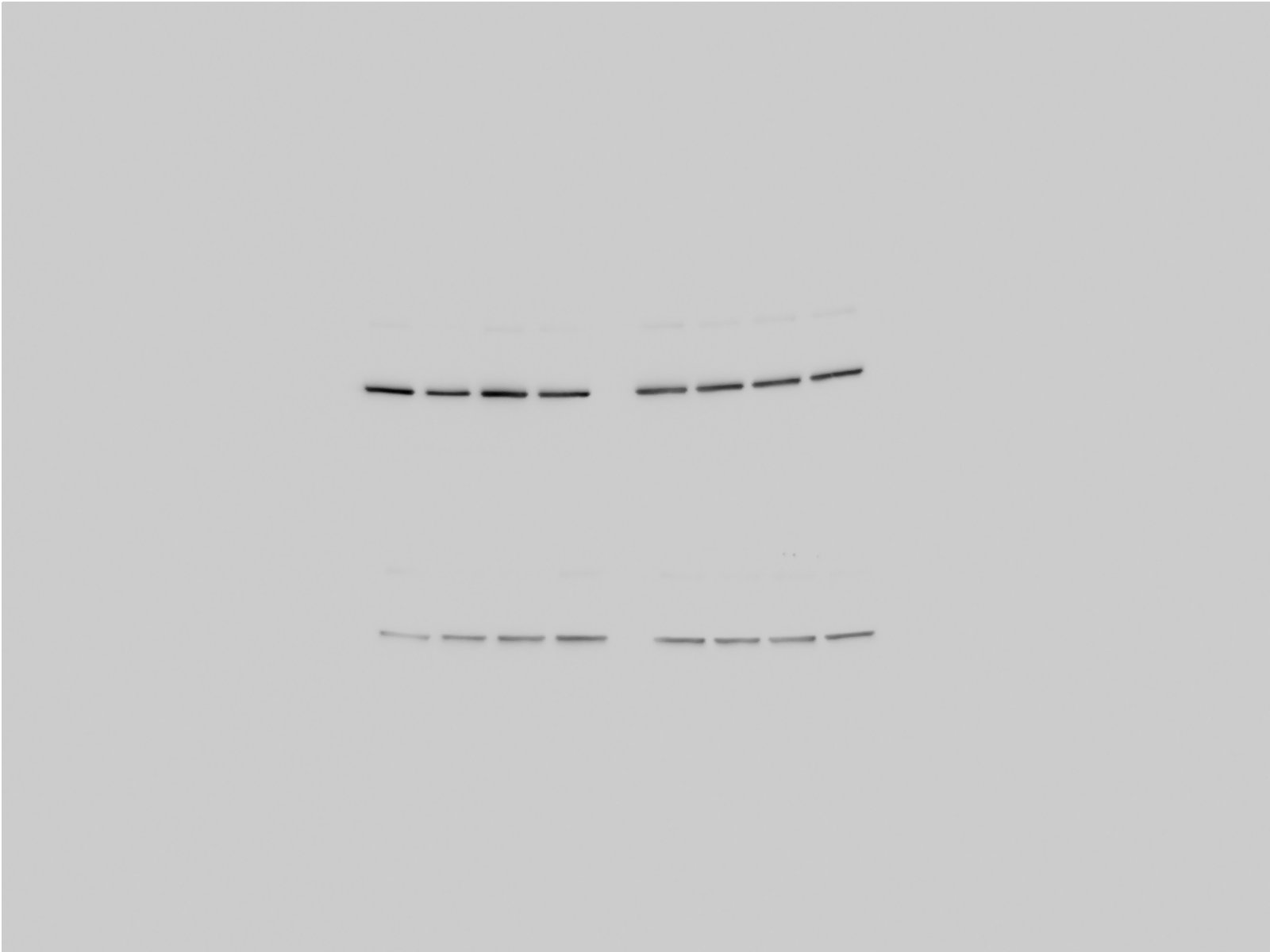

Supplement: Figure 2—figure supplement 1—source data 1. [file elife-90854-fig2-figsupp1-data1.zip › S4 B/Tubulin Gapdh 630_9810.jpg]

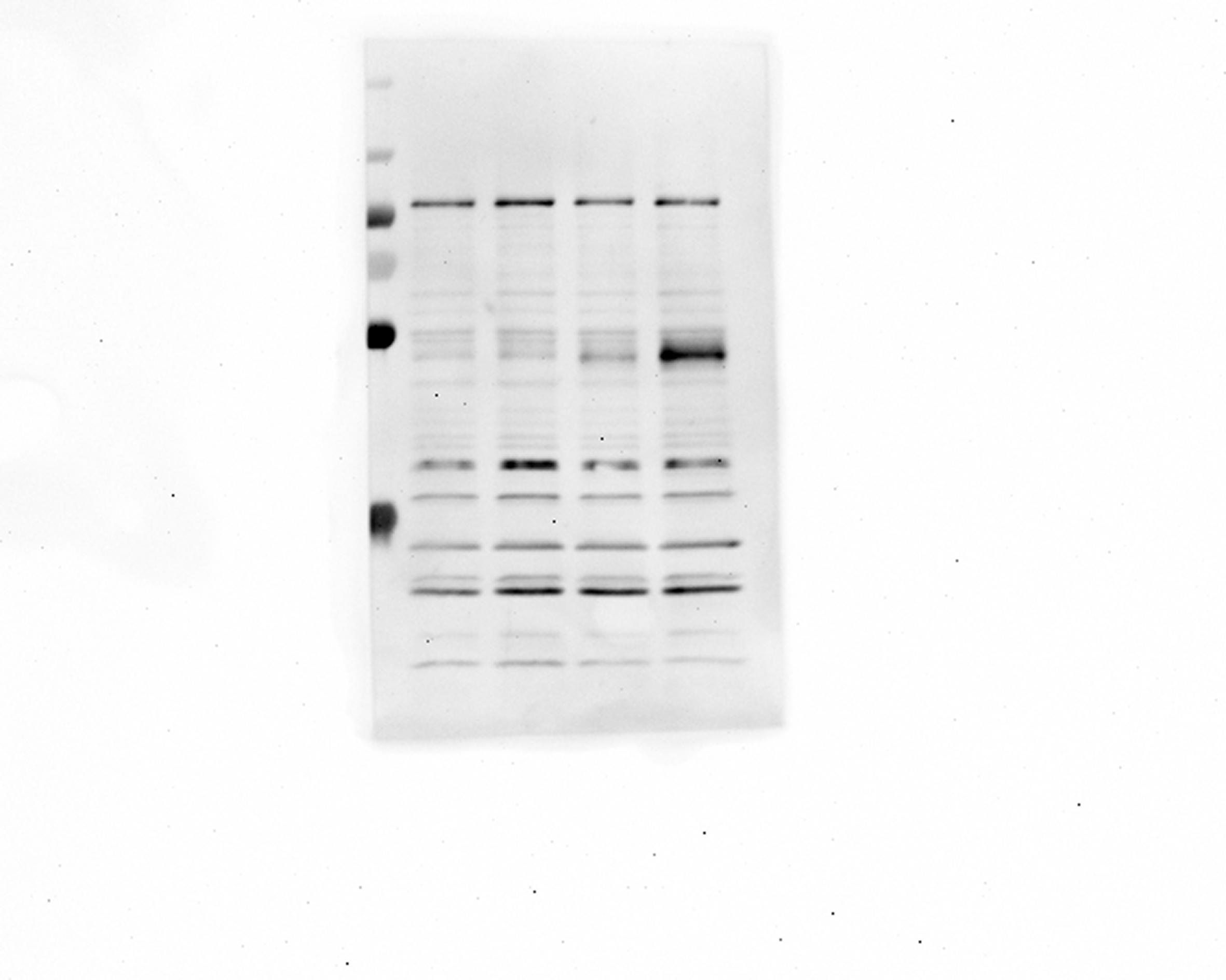

Supplement: Figure 3—source data 1. [file elife-90854-fig3-data1.zip › Fig 3 H/ATF4 CHEMI_03292023_113140.tif]

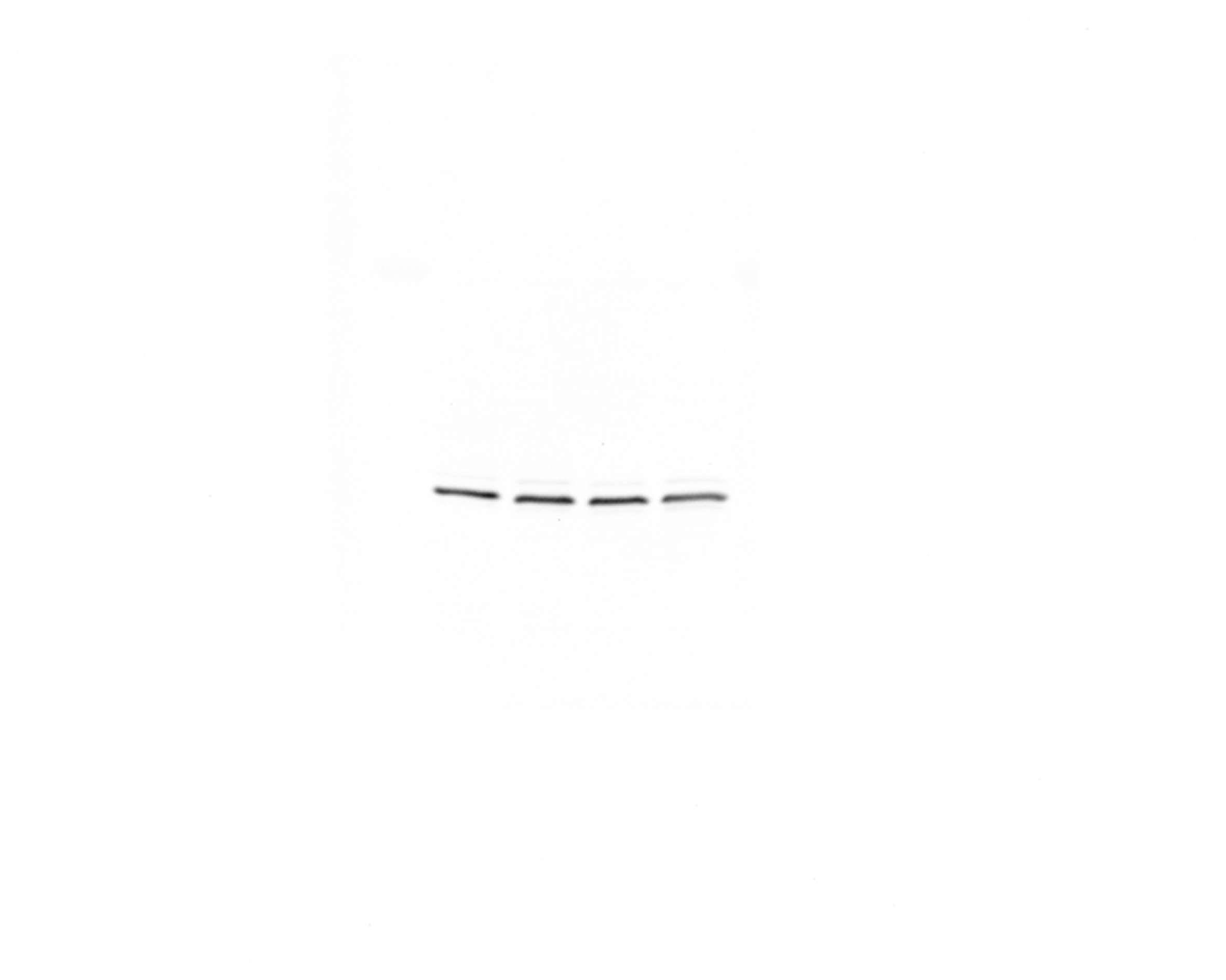

Supplement: Figure 3—source data 1. [file elife-90854-fig3-data1.zip › Fig 3 H/CHAC1 CHEMI_03292023_112437.tif]

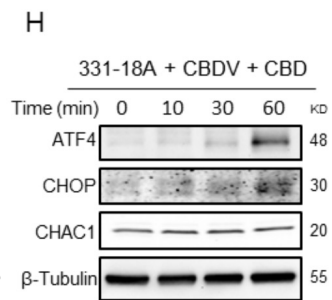

**ATF4**

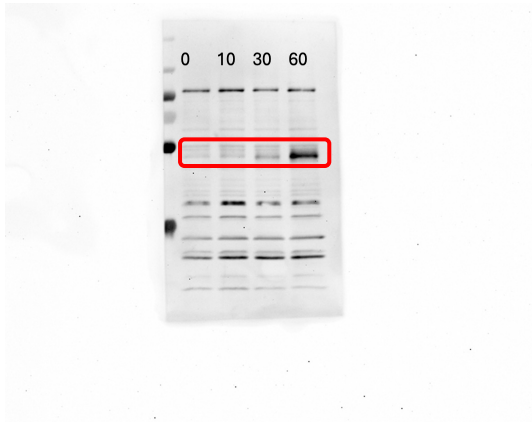

**CHOP**

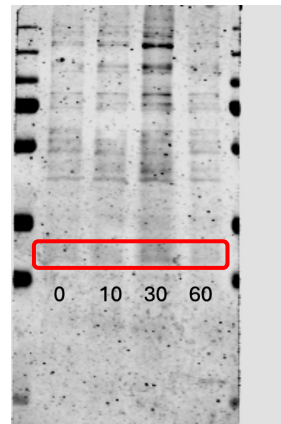

**CHAC1**

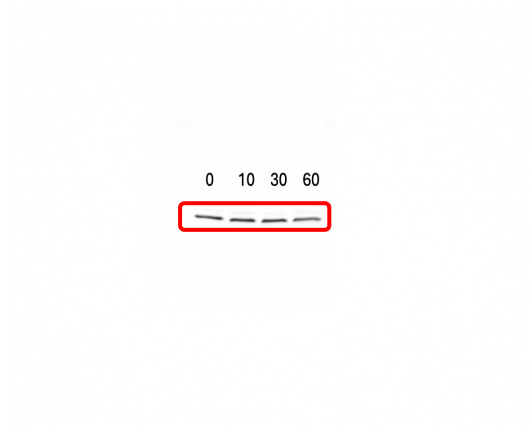

**$\beta$ -Tubulin**

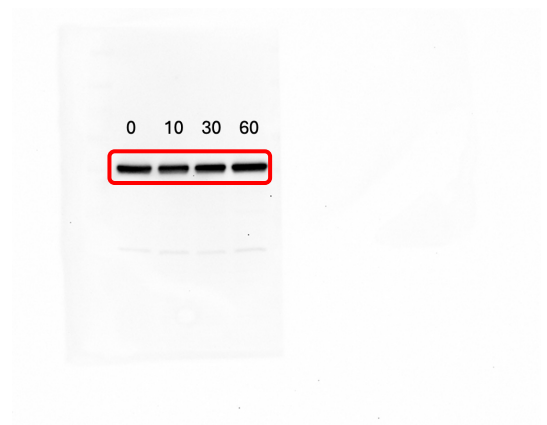

Supplement: Figure 3—source data 1. [file elife-90854-fig3-data1.zip › Fig 3 H/Figure 3 H.pdf]

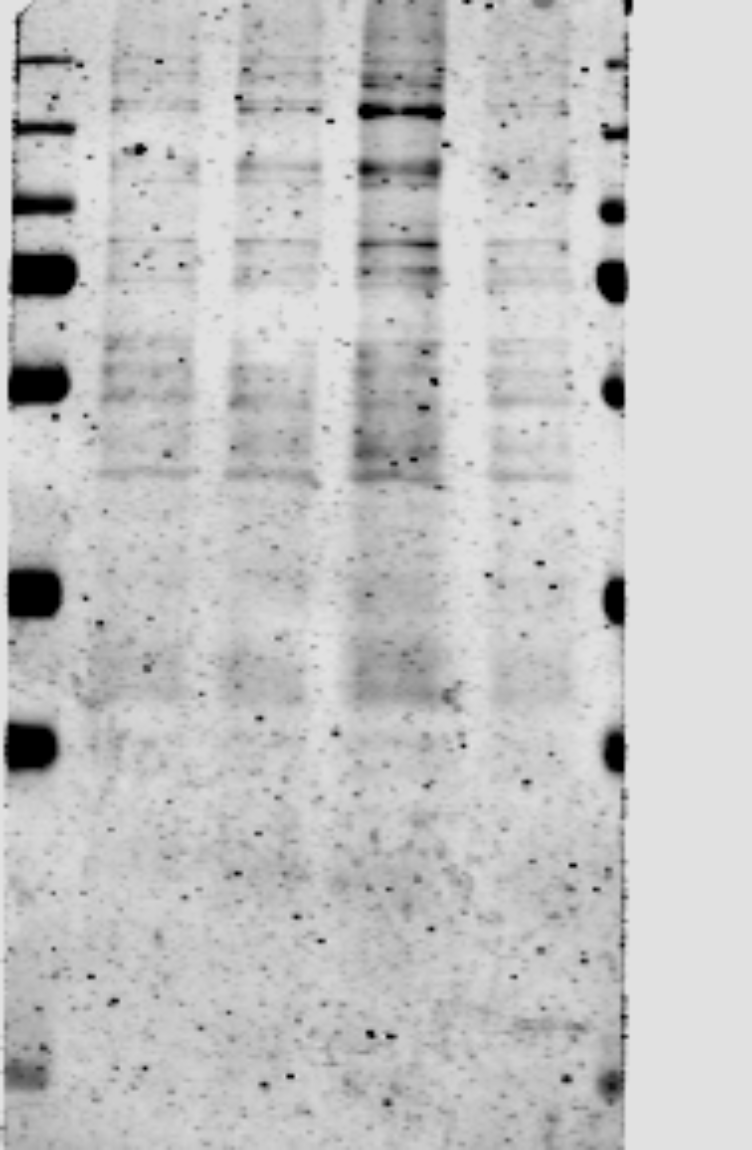

Supplement: Figure 3—source data 1. [file elife-90854-fig3-data1.zip › Fig 3 H/Molt-4 CHOP 0-10-30-60-3 Rb.tif]

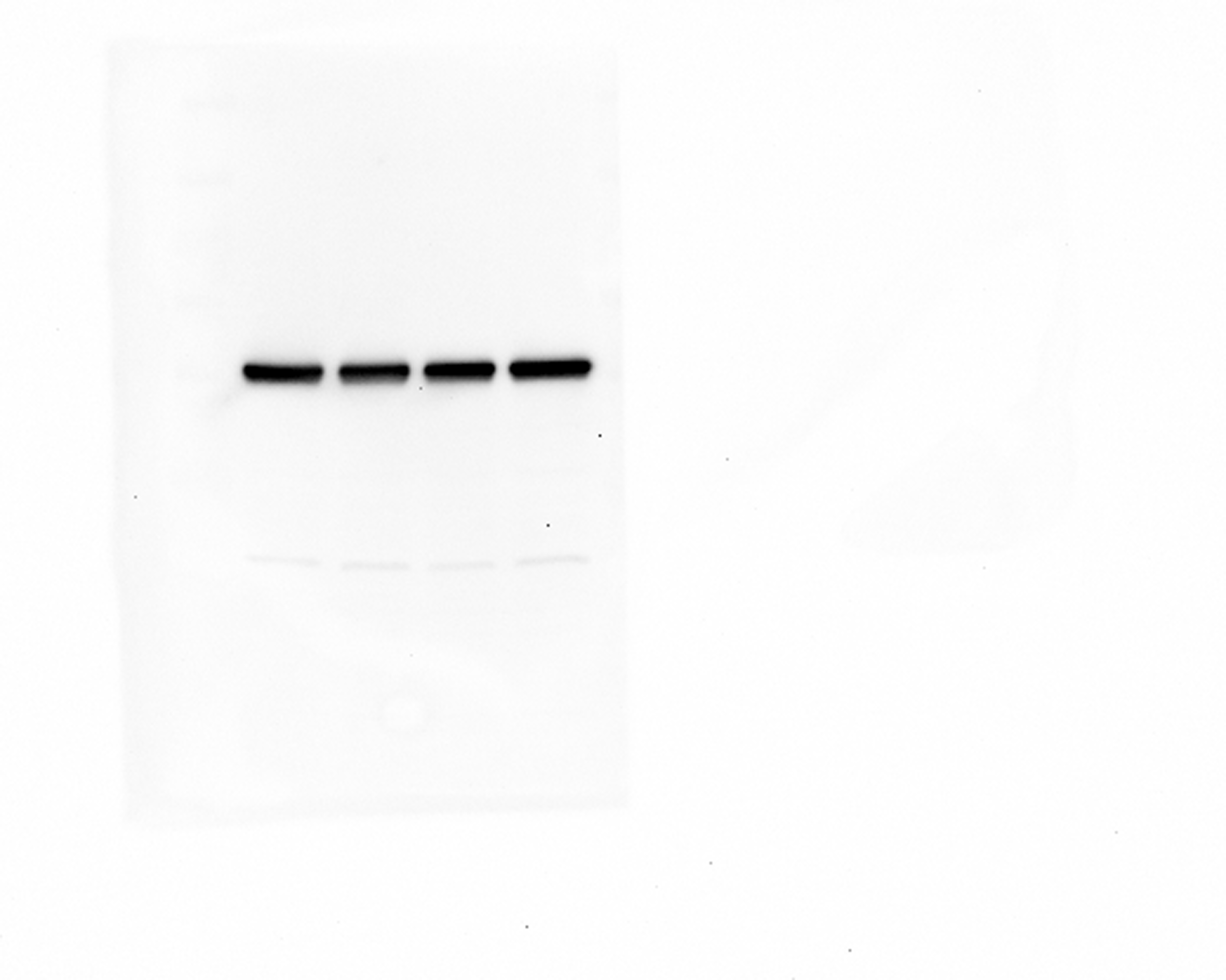

Supplement: Figure 3—source data 1. [file elife-90854-fig3-data1.zip › Fig 3 H/Tubulin CHEMI_03292023_144536.tif]

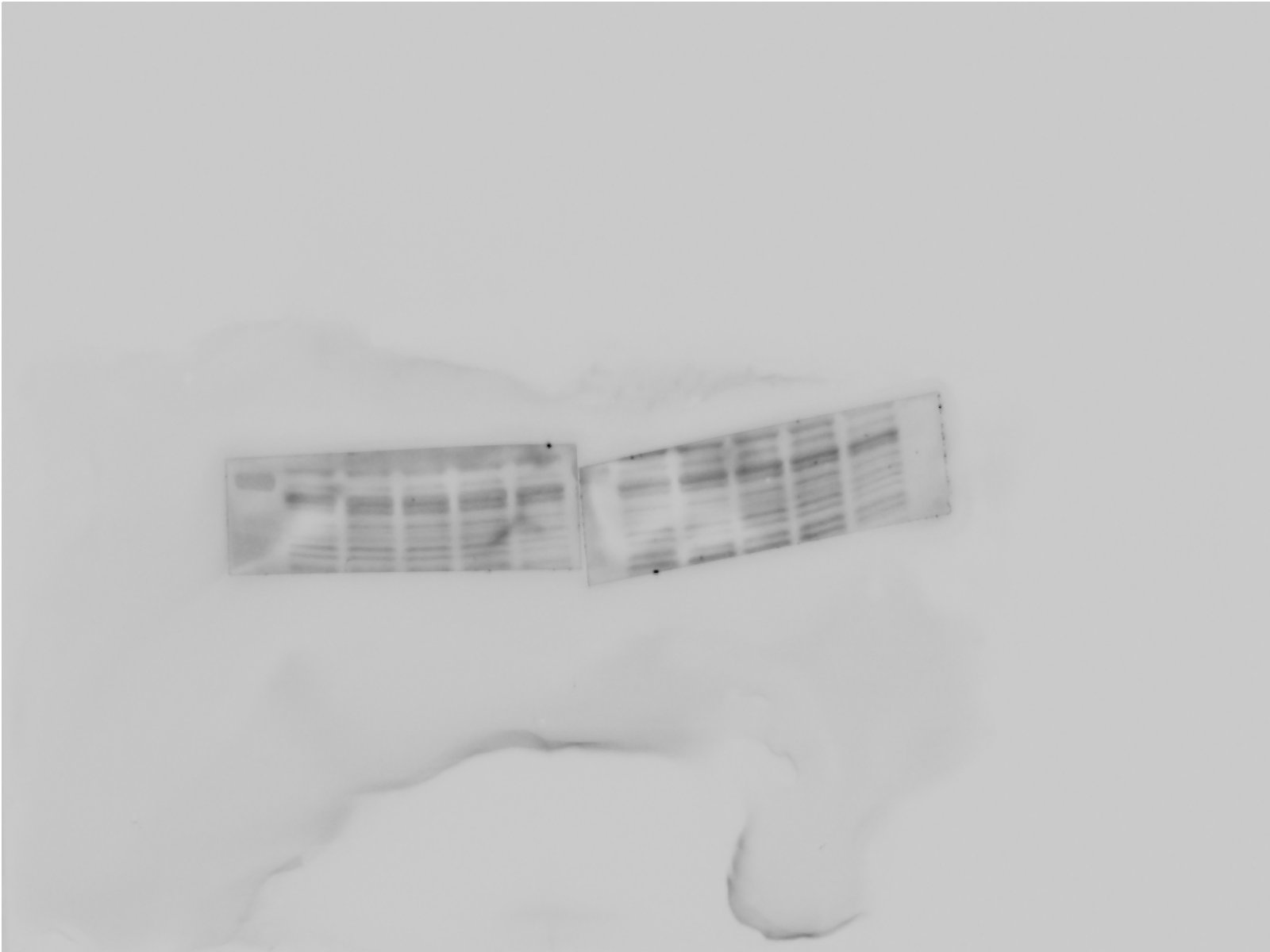

Supplement: Figure 3—figure supplement 1—source data 1. [file elife-90854-fig3-figsupp1-data1.zip › chac1 0-120.jpg]

I

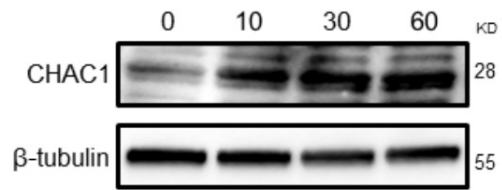

**CHAC1**

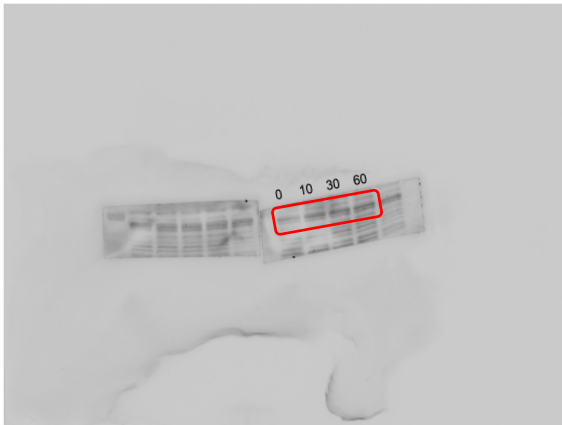

**B-Tubulin**

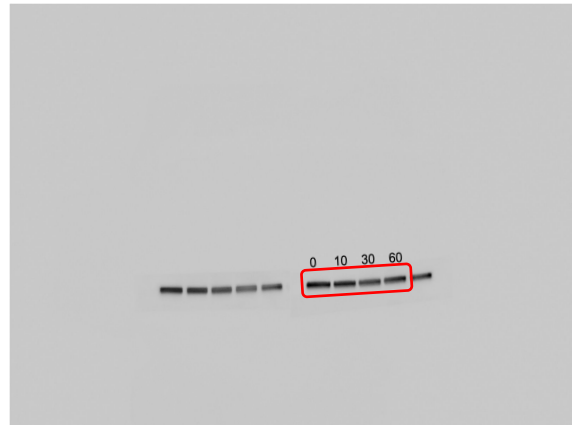

Supplement: Figure 3—figure supplement 1—source data 1. [file elife-90854-fig3-figsupp1-data1.zip › Figure S5 E.pdf]

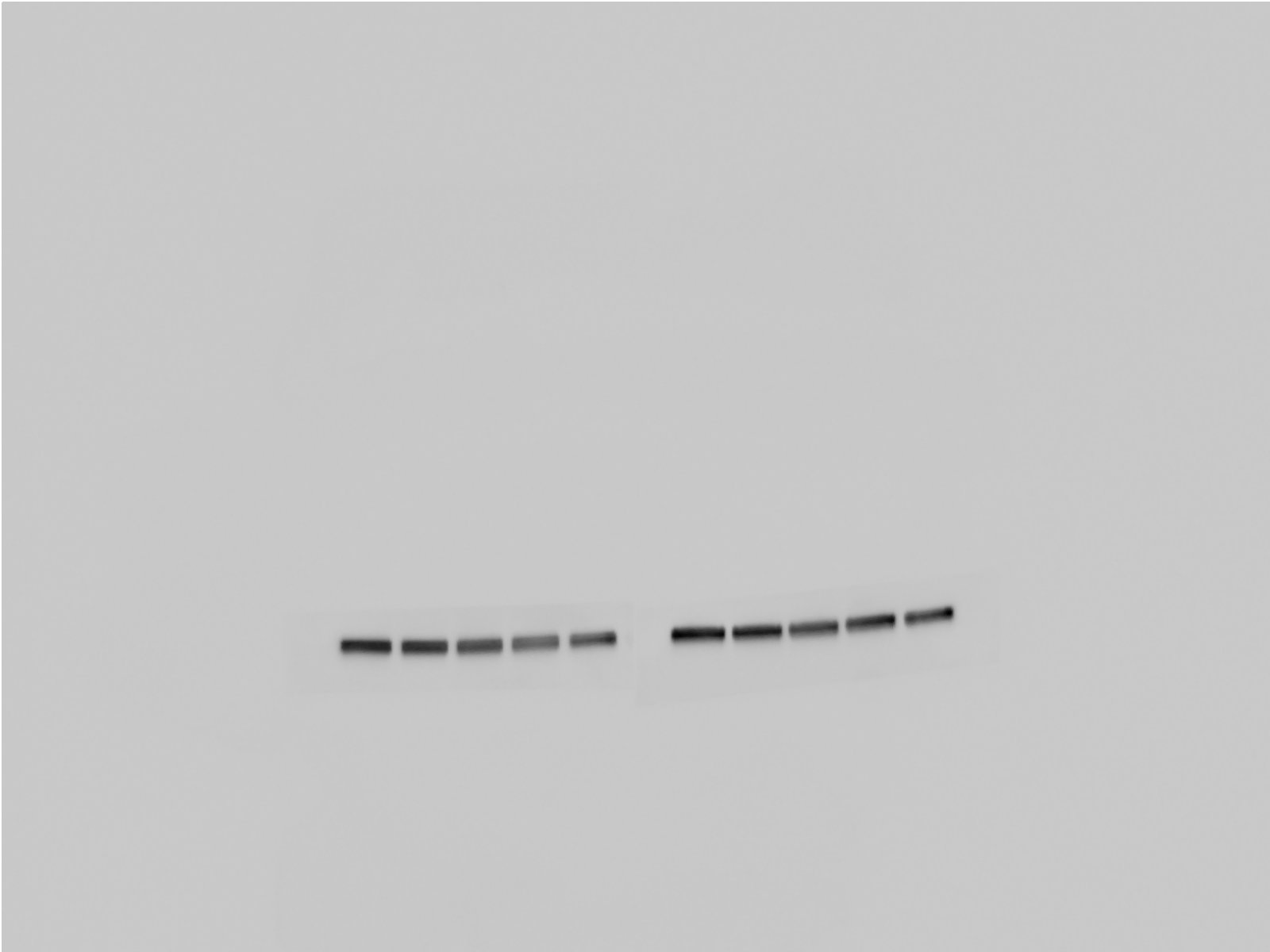

Supplement: Figure 3—figure supplement 1—source data 1. [file elife-90854-fig3-figsupp1-data1.zip › tubulin 0-120.jpg]

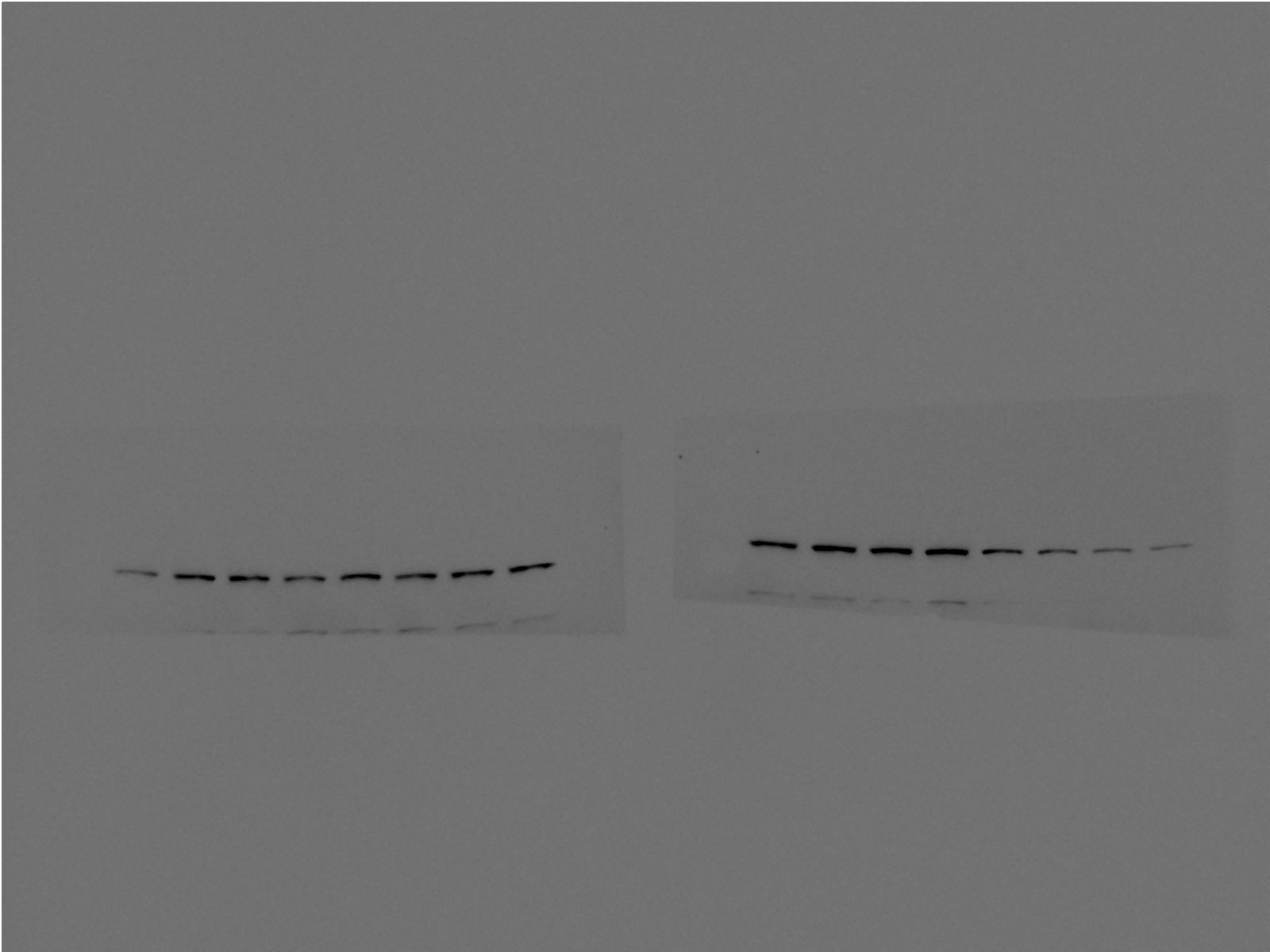

Supplement: Figure 3—figure supplement 1—source data 2. [file elife-90854-fig3-figsupp1-data2.zip › CB2 TRPV1 CHAC1.jpg]

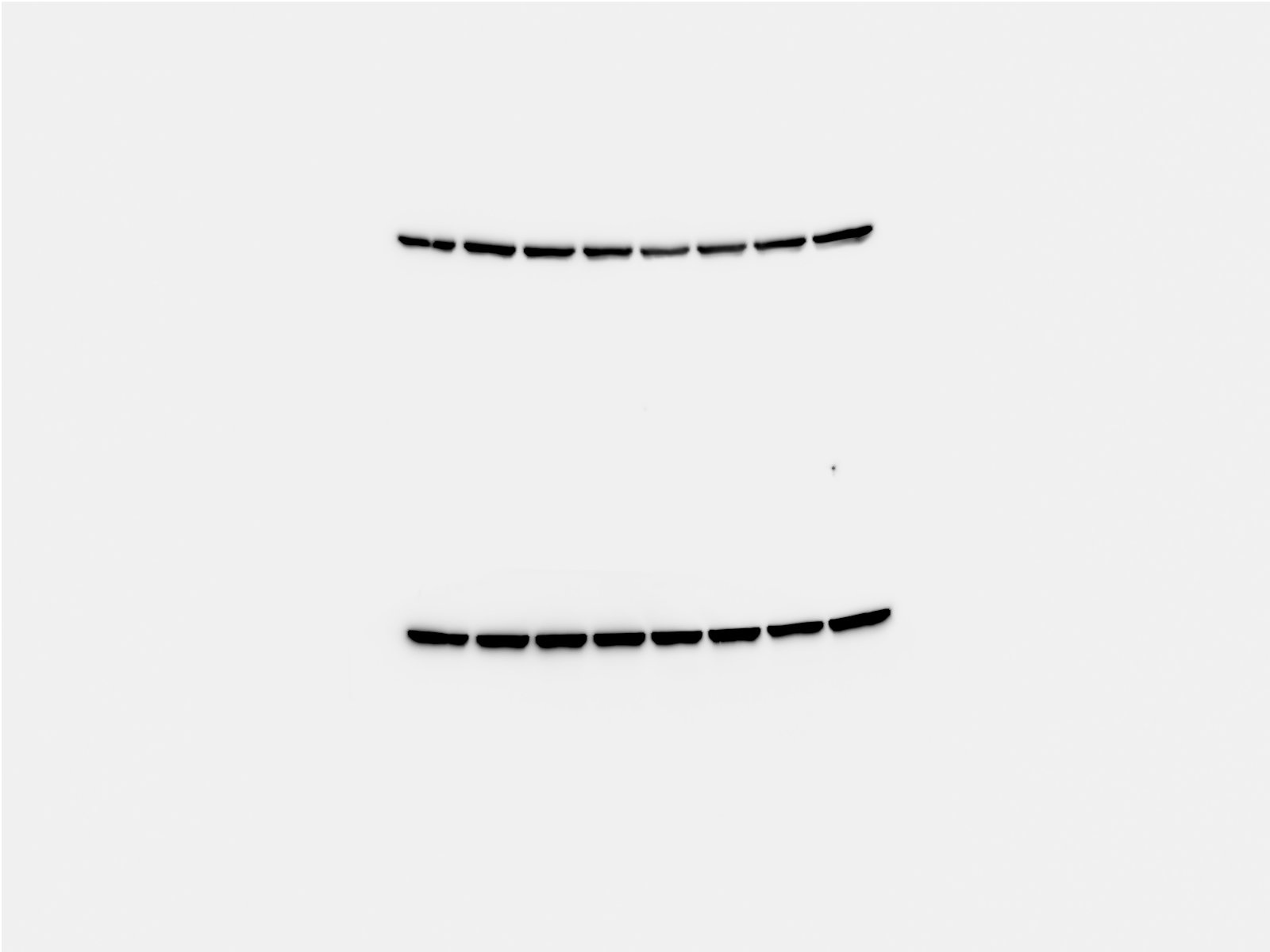

Supplement: Figure 3—figure supplement 1—source data 2. [file elife-90854-fig3-figsupp1-data2.zip › CB2 TRPV1 GAPDH.jpg]

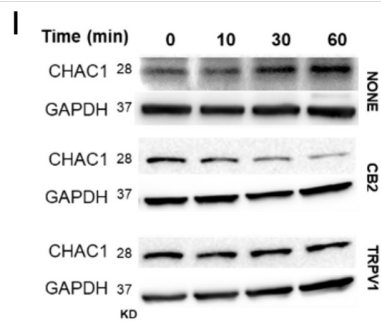

**CHAC1 - None**

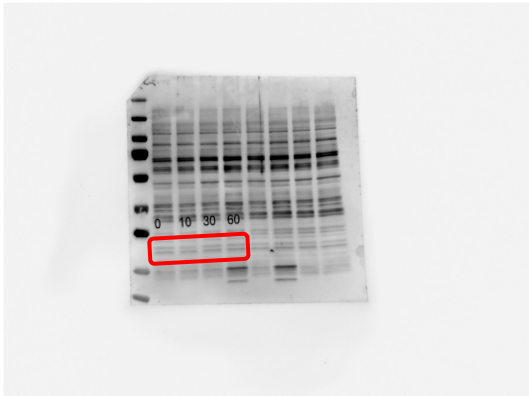

**GAPDH - None**

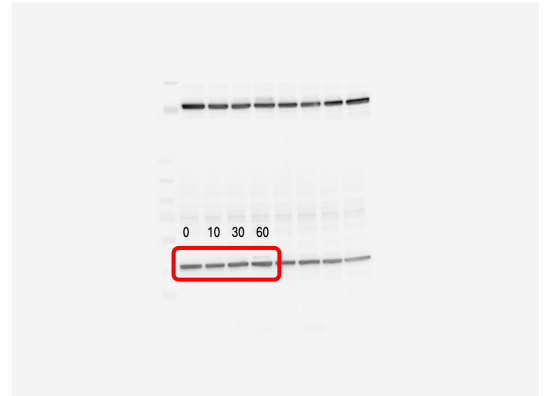

**CHAC1**

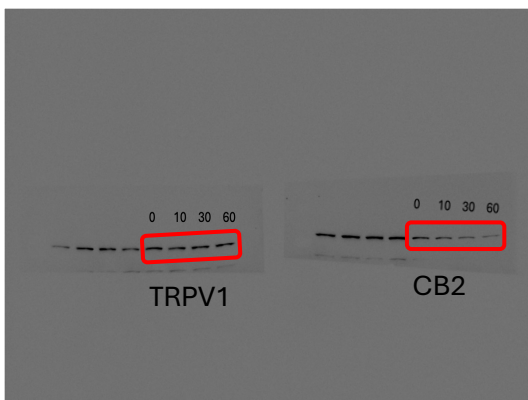

**GAPDH**

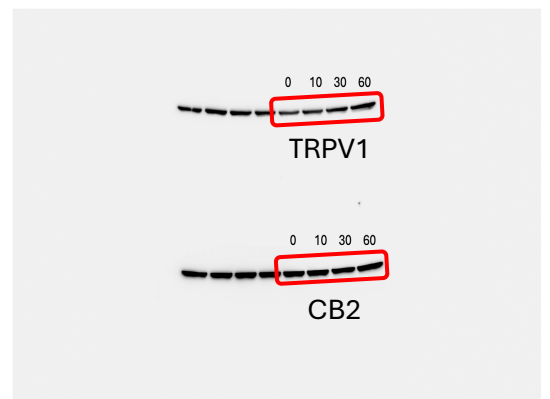

Supplement: Figure 3—figure supplement 1—source data 2. [file elife-90854-fig3-figsupp1-data2.zip › Figure 3S1SD2.pdf]

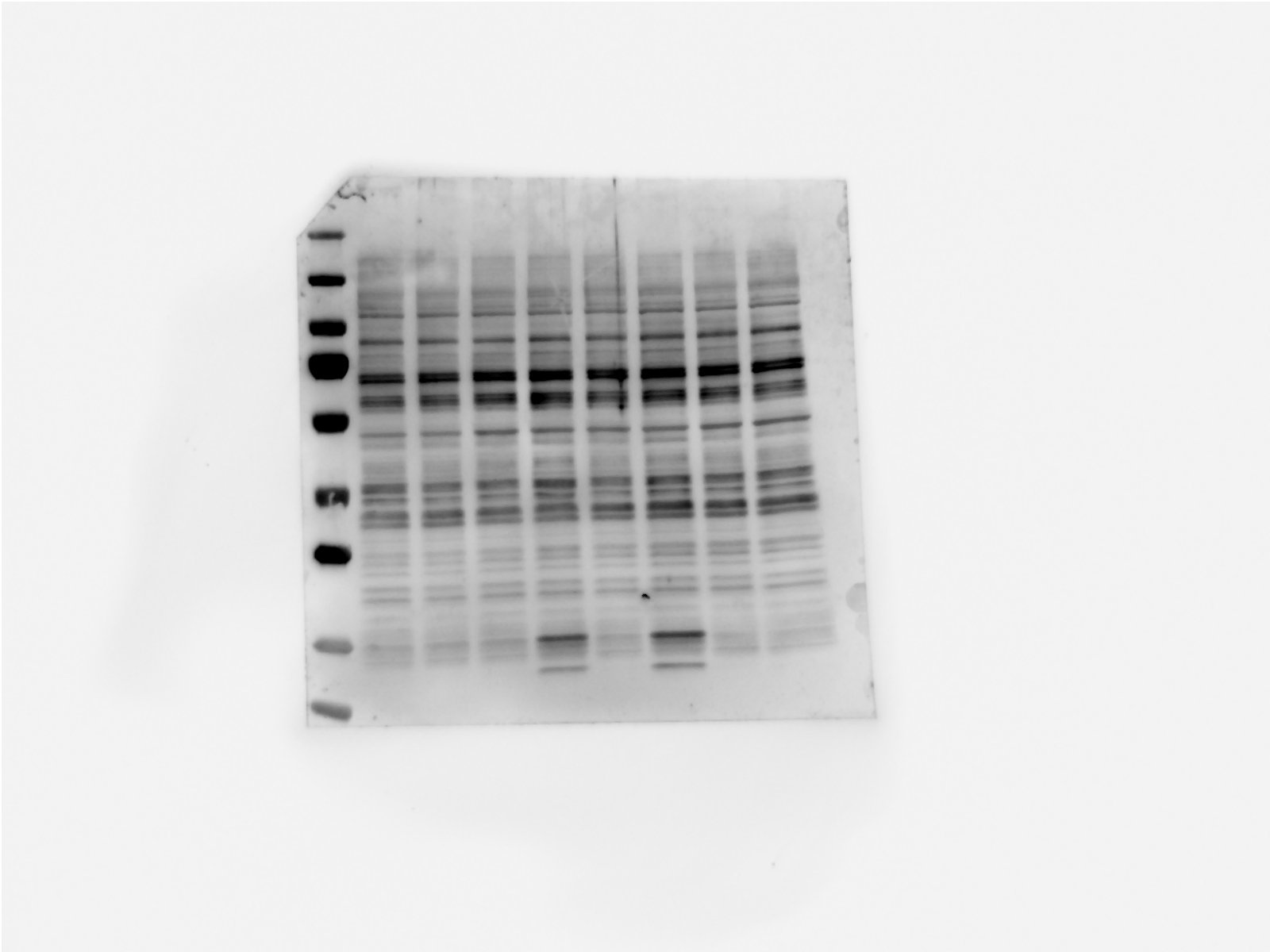

Supplement: Figure 3—figure supplement 1—source data 2. [file elife-90854-fig3-figsupp1-data2.zip › None chac1 trpv1.jpg]

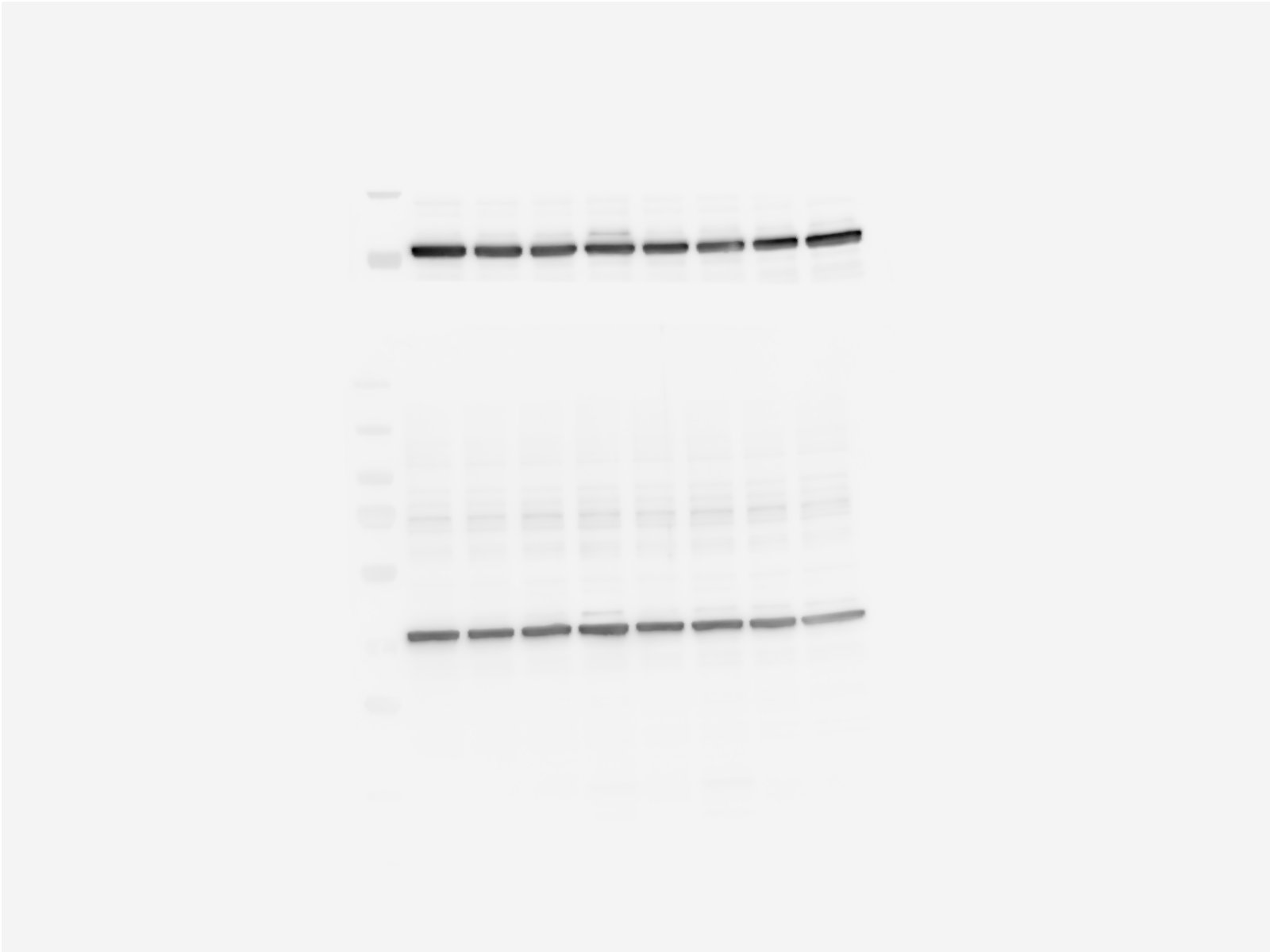

Supplement: Figure 3—figure supplement 1—source data 2. [file elife-90854-fig3-figsupp1-data2.zip › None gapdh cb2_trpv1.jpg]

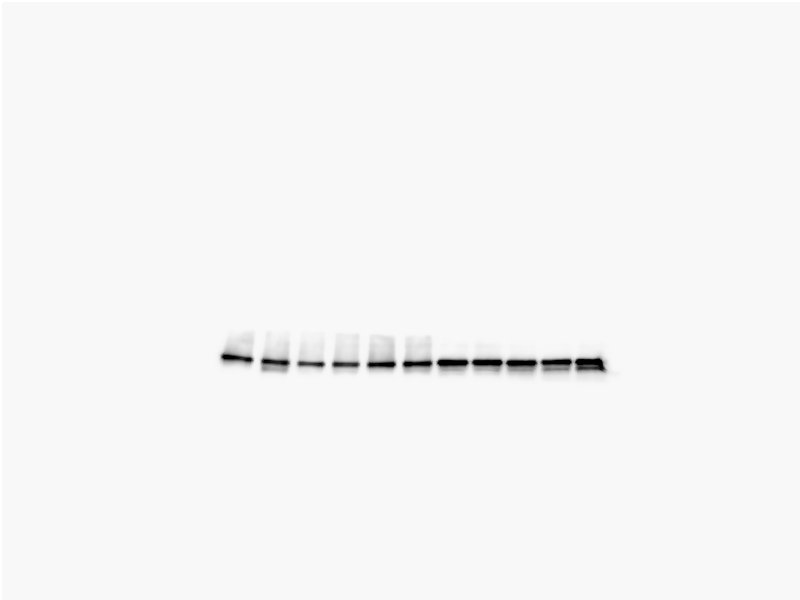

Supplement: Figure 4—source data 1. [file elife-90854-fig4-data1.zip › Fig 4 C/eIF2a M D_3X NONE_IRISB_SET2_Z-VAD_CID_PTX 19.1.jpg]

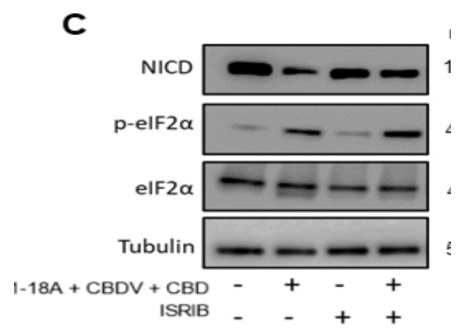

**NICD**

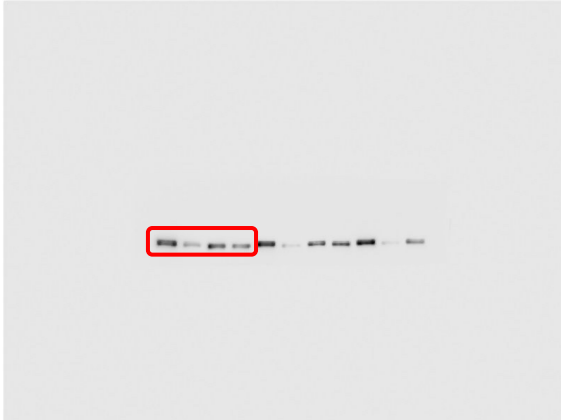

**P-eIF2α**

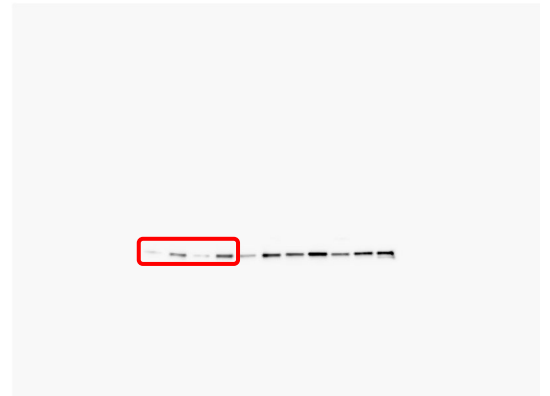

**eIF2α**

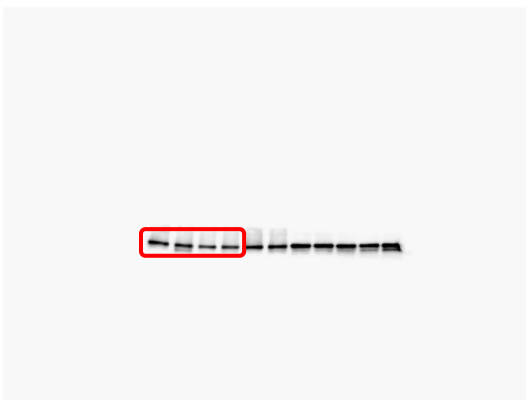

**β-Tubulin**

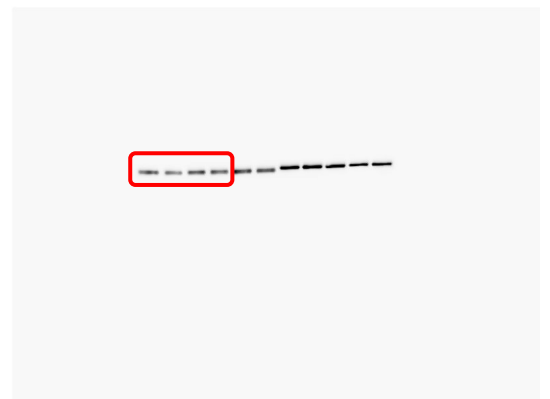

Supplement: Figure 4—source data 1. [file elife-90854-fig4-data1.zip › Fig 4 C/Figure 4 C.pdf]

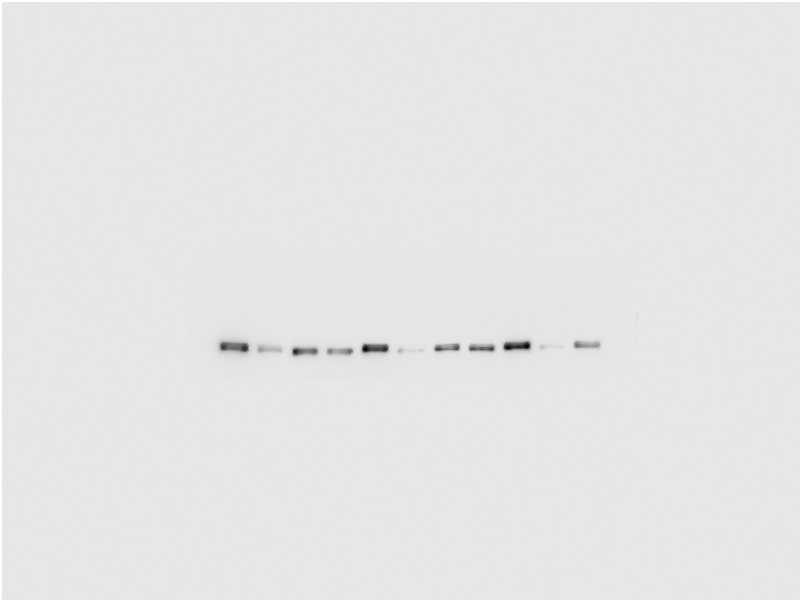

Supplement: Figure 4—source data 1. [file elife-90854-fig4-data1.zip › Fig 4 C/NICD M D_3X NONE_IRISB_SET2_Z-VAD_CID_PTX 19.1.jpg]

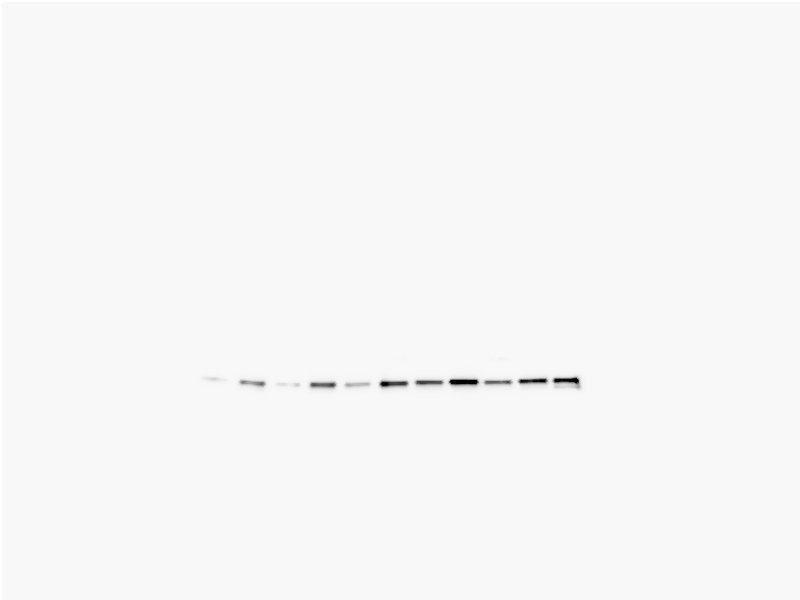

Supplement: Figure 4—source data 1. [file elife-90854-fig4-data1.zip › Fig 4 C/p-eIF2a M D_3X NONE_IRISB_SET2_Z-VAD_CID_PTX 19.1.jpg]

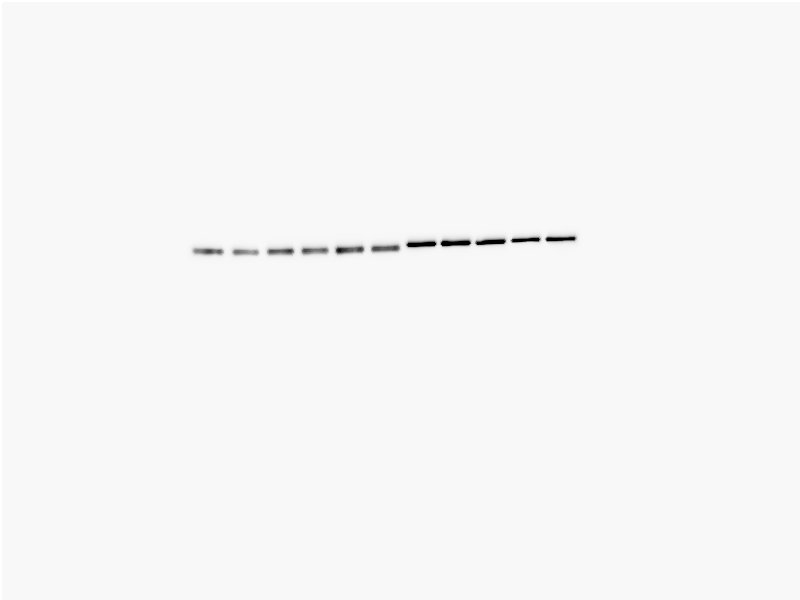

Supplement: Figure 4—source data 1. [file elife-90854-fig4-data1.zip › Fig 4 C/Tubulin M D_3X NONE_IRISB_SET2_Z-VAD_CID_PTX 19.1.jpg]

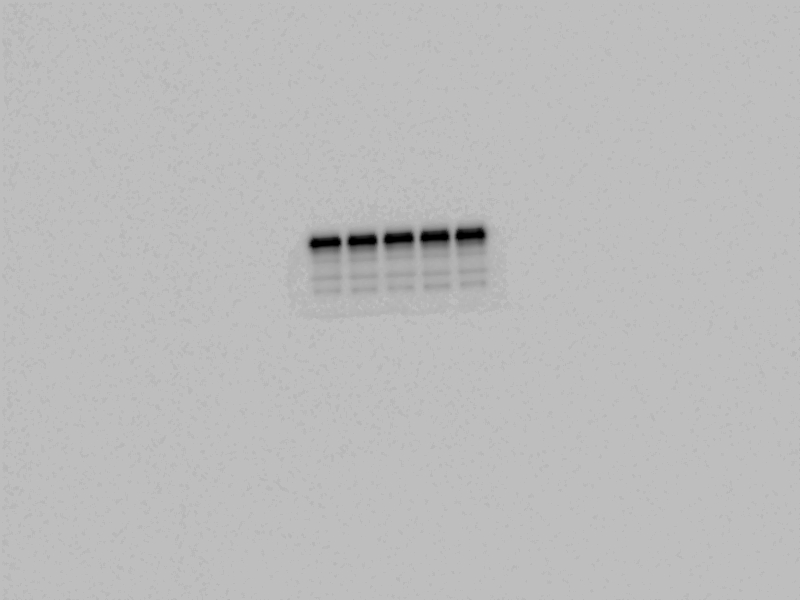

Supplement: Figure 4—figure supplement 1—source data 1. [file elife-90854-fig4-figsupp1-data1.zip › Fig S6 B/eIF2a M 0-120 14.12.jpg]

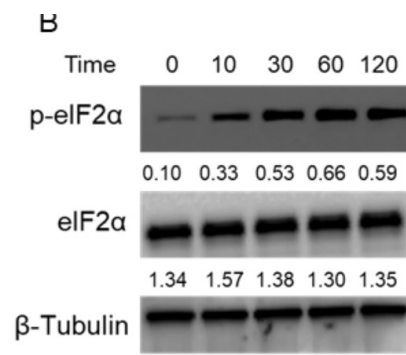

## P-eIF2 $\alpha$

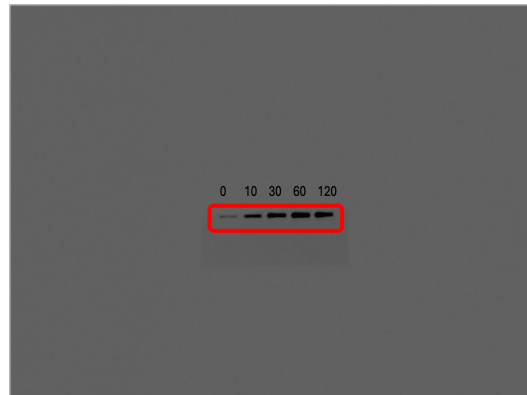

## eIF2 $\alpha$

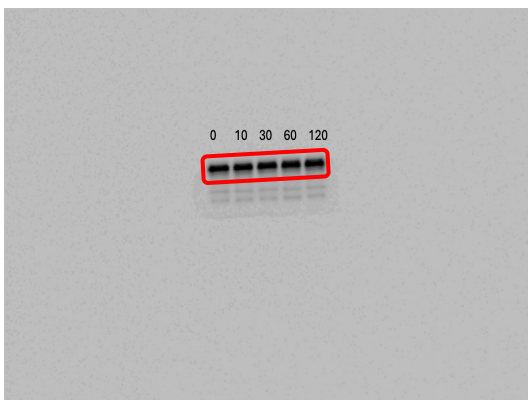

## $\beta$ -Tubulin

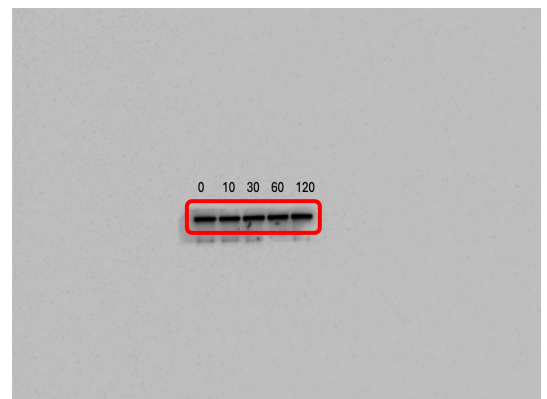

Supplement: Figure 4—figure supplement 1—source data 1. [file elife-90854-fig4-figsupp1-data1.zip › Fig S6 B/Figure S6 B.pdf]

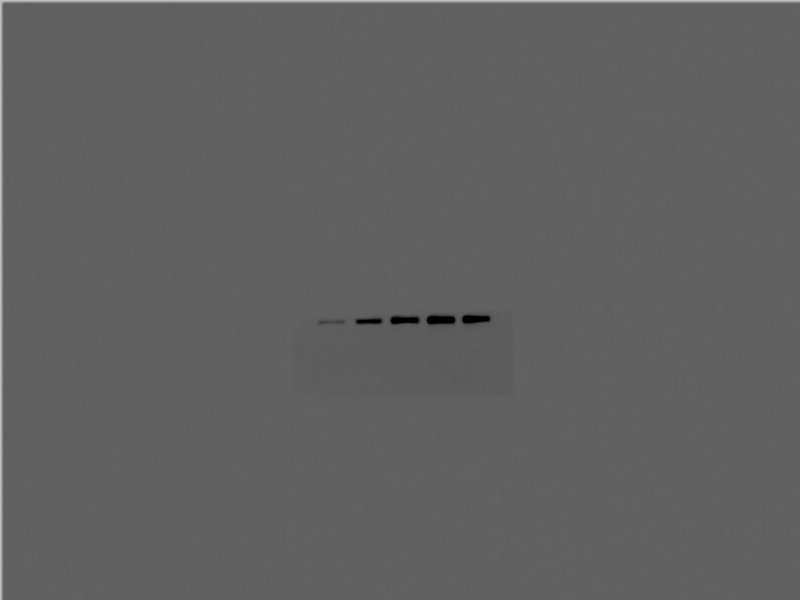

Supplement: Figure 4—figure supplement 1—source data 1. [file elife-90854-fig4-figsupp1-data1.zip › Fig S6 B/p-eIF2a M 0-120 14.12.jpg]

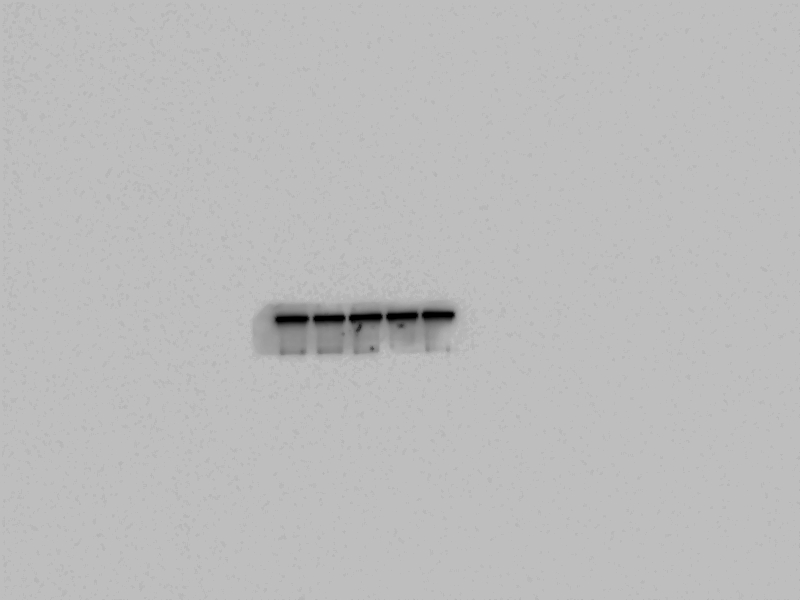

Supplement: Figure 4—figure supplement 1—source data 1. [file elife-90854-fig4-figsupp1-data1.zip › Fig S6 B/tubulin M 0-120 14.12.jpg]

E

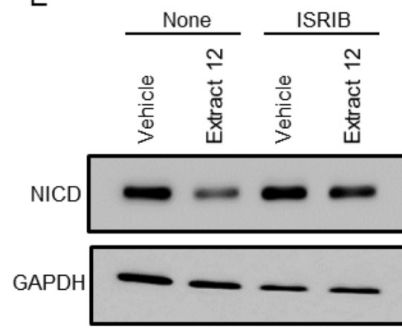

NICD

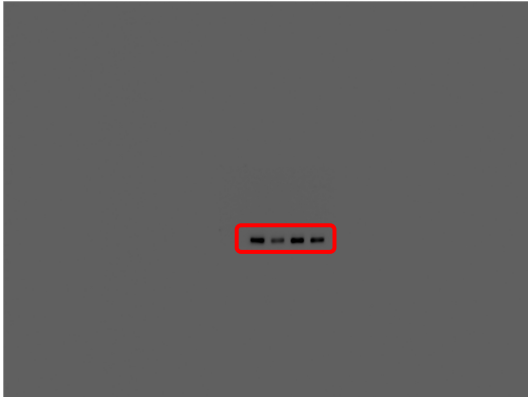

GAPDH

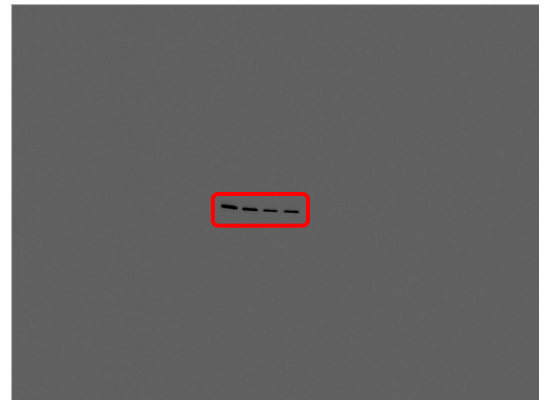

Supplement: Figure 4—figure supplement 1—source data 2. [file elife-90854-fig4-figsupp1-data2.zip › Fig S6 E/Figure S6 E.pdf]

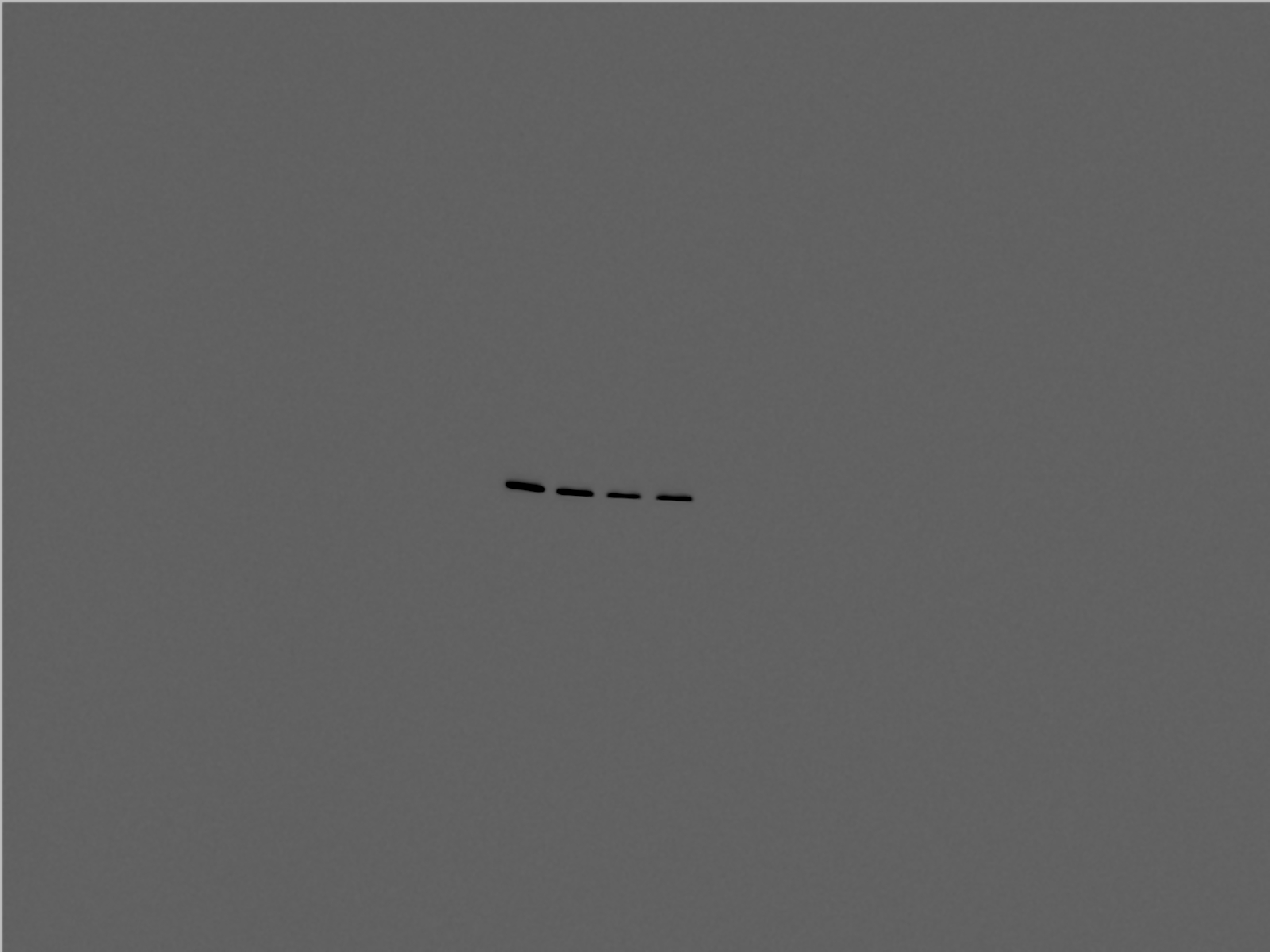

Supplement: Figure 4—figure supplement 1—source data 2. [file elife-90854-fig4-figsupp1-data2.zip › Fig S6 E/GAPDH ISRIB 16.2.jpg]

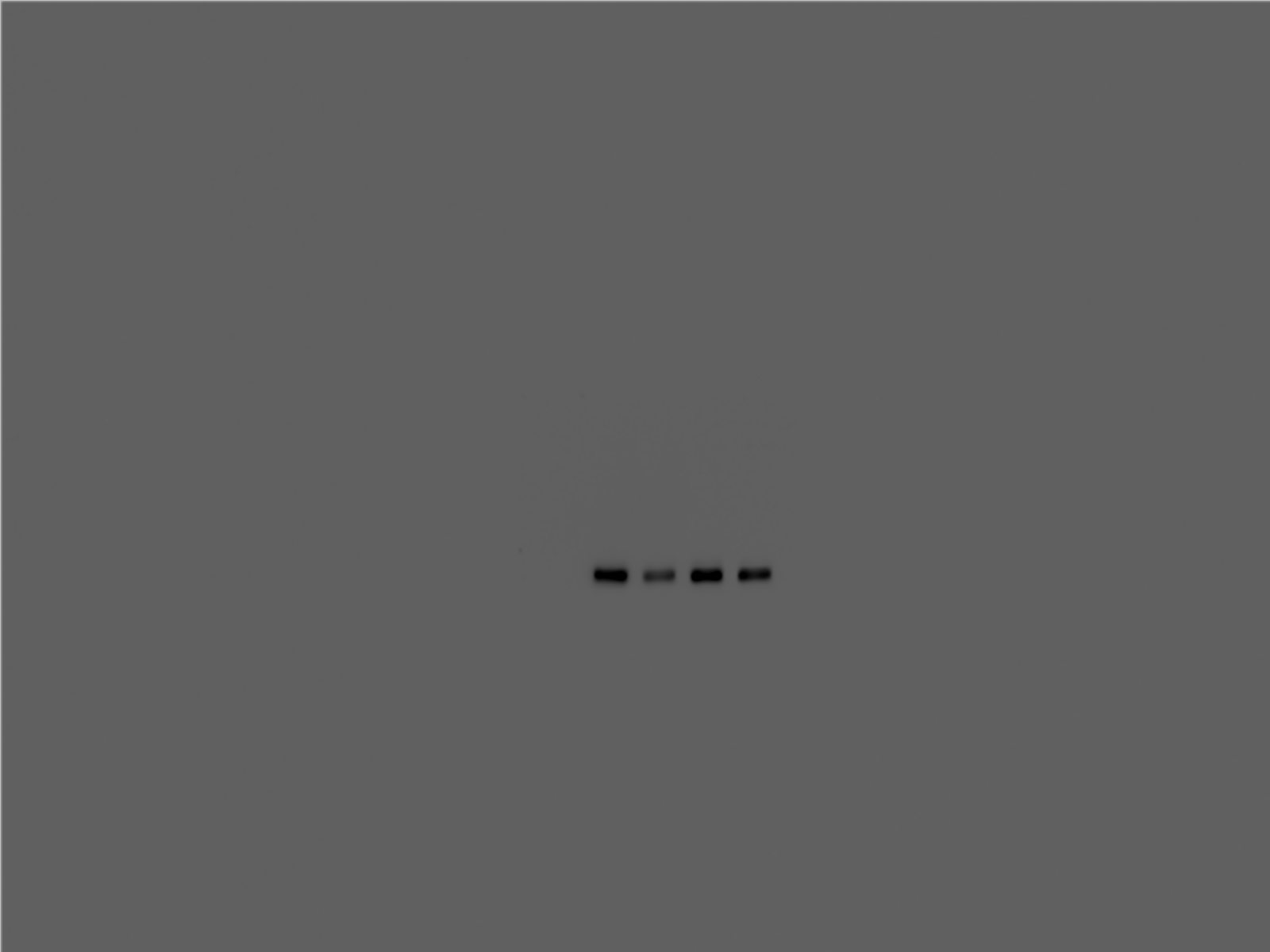

Supplement: Figure 4—figure supplement 1—source data 2. [file elife-90854-fig4-figsupp1-data2.zip › Fig S6 E/NICD ISRIB 16.2.jpg]

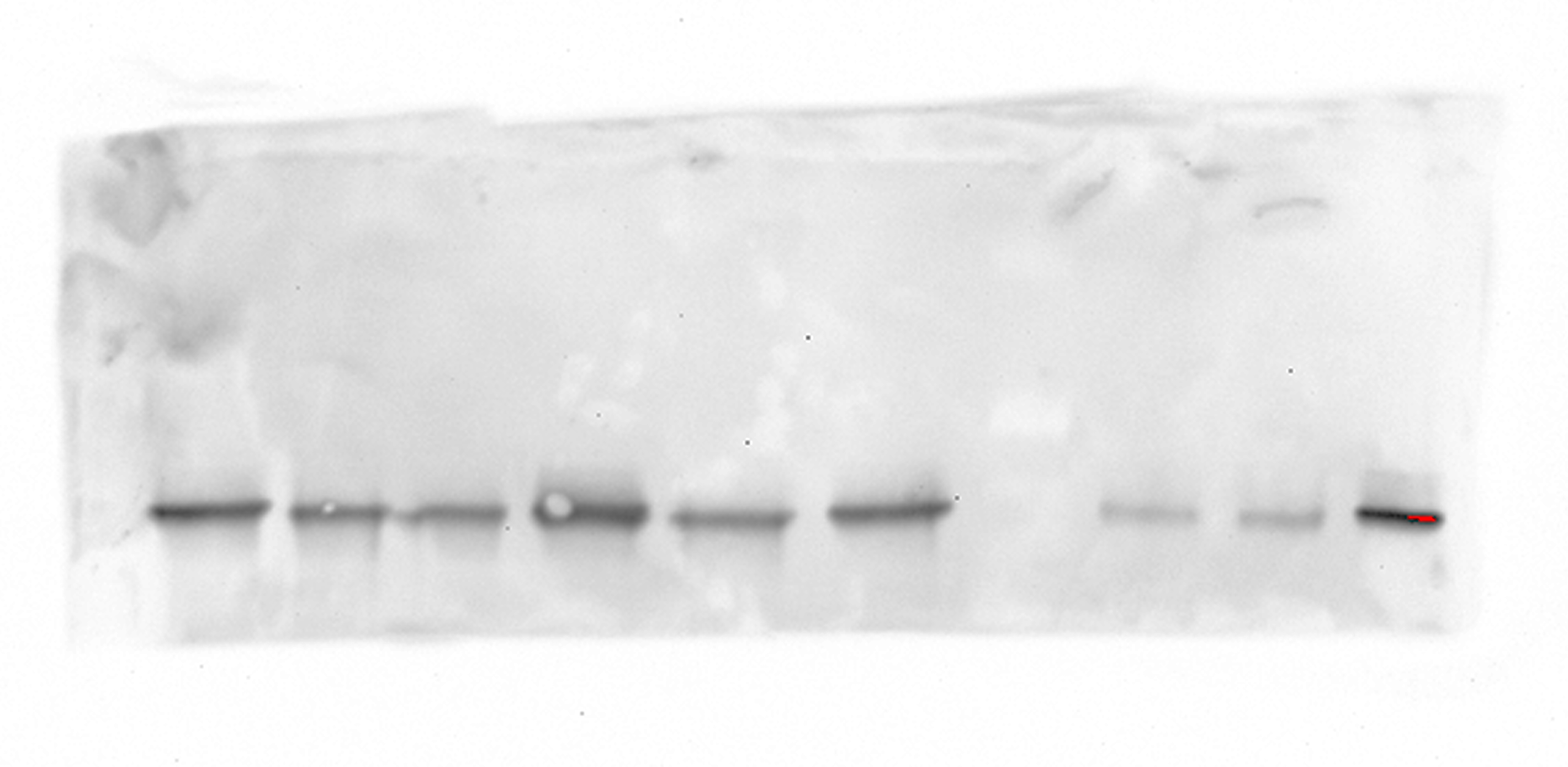

Supplement: Figure 6—source data 1. [file elife-90854-fig6-data1.zip › Tumor WB GAPDH.tif]

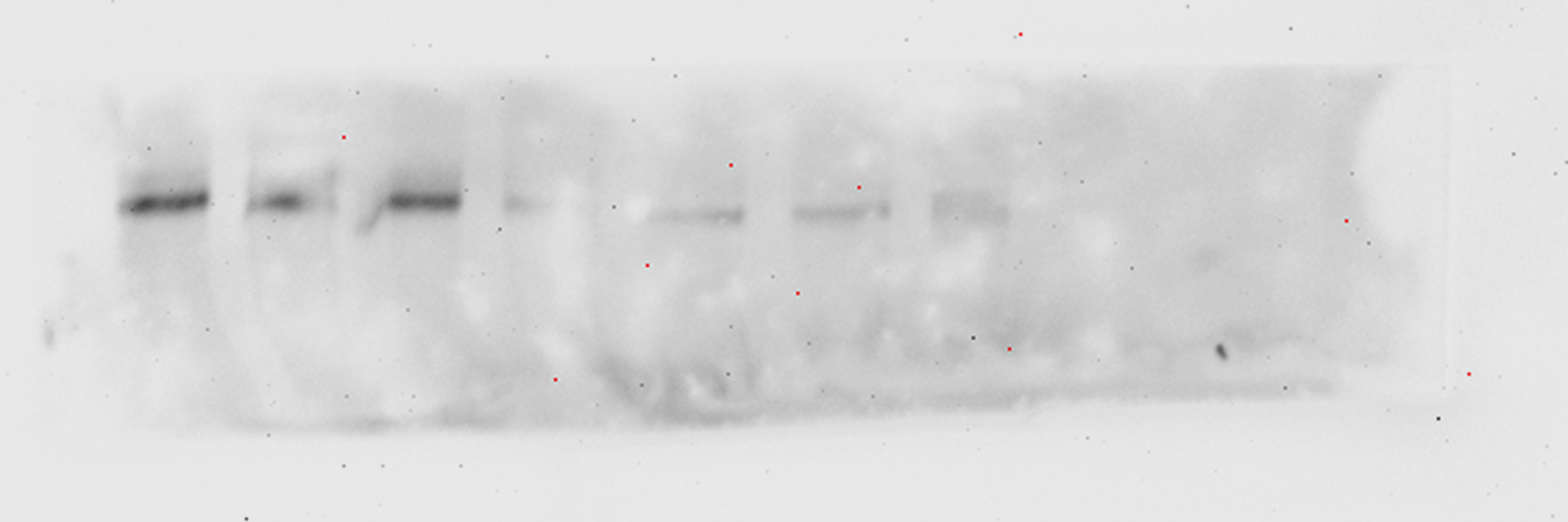

Supplement: Figure 6—source data 1. [file elife-90854-fig6-data1.zip › Tumor WB NICD.png]
