# Supplementary material for: Cannabinoid combination targets NOTCH1-mutated T-cell acute lymphoblastic leukemia through the integrated stress response pathway
Source: eLife. 2024 Sep 11;12:RP90854. doi: 10.7554/eLife.90854 (PMC11390110; doi:10.7554/eLife.90854)
Supplement: Figure 6—source data 1. [file elife-90854-fig6-data1.zip › Fig6 - source data 1.pptx]

## Slide 1
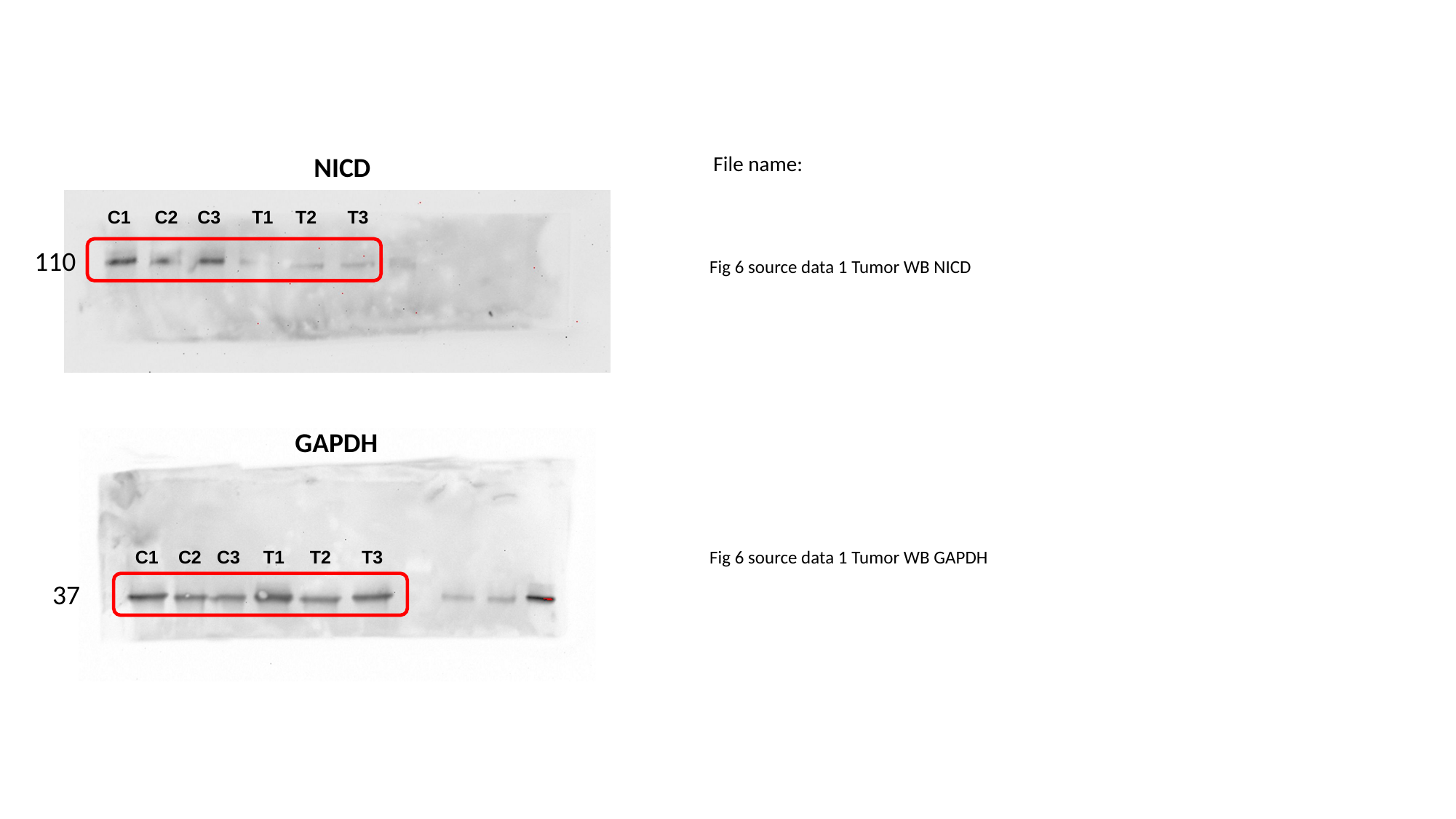

NICD
File name:
C1
C2
C3
T1
T2
T3
110
Fig 6 source data 1 Tumor WB NICD
GAPDH
C1
C2
C3
T1
T2
T3
Fig 6 source data 1 Tumor WB GAPDH
37
