# Supplementary material for: Cannabinoid combination targets NOTCH1-mutated T-cell acute lymphoblastic leukemia through the integrated stress response pathway
Source: eLife. 2024 Sep 11;12:RP90854. doi: 10.7554/eLife.90854 (PMC11390110; doi:10.7554/eLife.90854)
Supplement: Supplementary file 1. [file elife-90854-supp1.docx]

Supplementary file 1. Phytocannabinoid concentrations by UHPLC/LC-MS of fractions relative to the whole extract

|  |  | |  | |  |  |  |
| --- | --- | --- | --- | --- | --- | --- | --- |
|  |  | | **Fraction** | | | | |
| **[%w/w]** | **Whole  extract** | **1** | | **2** | | **3** | **4** |
| **CBC** | 2.856 | 0.000 | | 0.000 | | 32.607 | 0.099 |
| **CBCA** | 0.012 | 0.000 | | 0.000 | | 0.001 | 0.153 |
| **CBCA-C4** | 0.000 | 0.000 | | 0.000 | | 0.001 | 0.000 |
| **CBC-C4** | 0.023 | 0.000 | | 0.000 | | 0.248 | 0.000 |
| **CBCMA** | 0.004 | 0.000 | | 0.003 | | 0.004 | 0.000 |
| **CBCO** | 0.002 | 0.000 | | 0.003 | | 0.000 | 0.000 |
| **CBCOA** | 0.000 | 0.000 | | 0.000 | | 0.000 | 0.000 |
| **CBCV** | 0.267 | 0.000 | | 0.000 | | 3.028 | 0.000 |
| **CBCVA** | 0.002 | 0.000 | | 0.000 | | 0.008 | 0.000 |
| **CBD** | 53.384 | 0.018 | | 77.534 | | 0.045 | 0.034 |
| **CBDA** | 1.338 | 0.059 | | 1.800 | | 0.187 | 0.298 |
| **CBDA-C4** | 0.006 | 0.001 | | 0.009 | | 0.002 | 0.001 |
| **CBD-C4** | 0.256 | 0.000 | | 0.396 | | 0.000 | 0.000 |
| **CBDM** | 0.014 | 0.000 | | 0.005 | | 0.138 | 0.000 |
| **CBDMA** | 0.000 | 0.000 | | 0.000 | | 0.000 | 0.000 |
| **CBDO** | 0.004 | 0.004 | | 0.007 | | 0.000 | 0.000 |
| **CBDOA** | 0.000 | 0.001 | | 0.001 | | 0.000 | 0.000 |
| **CBDV** | 2.929 | 0.000 | | 4.416 | | 0.000 | 0.000 |
| **CBDVA** | 0.055 | 0.003 | | 0.084 | | 0.013 | 0.006 |
| **CBE** | 0.339 | 0.050 | | 0.544 | | 0.000 | 0.000 |
| **CBEA** | 0.010 | 0.001 | | 0.014 | | 0.002 | 0.002 |
| **CBEV** | 0.014 | 0.005 | | 0.020 | | 0.000 | 0.000 |
| **CBEVA** | 0.001 | 0.003 | | 0.001 | | 0.000 | 0.000 |
| **CBG** | 0.990 | 0.000 | | 1.544 | | 0.000 | 0.000 |
| **CBGA** | 0.024 | 0.000 | | 0.032 | | 0.004 | 0.007 |
| **CBGA-C4** | 0.000 | 0.000 | | 0.000 | | 0.000 | 0.000 |
| **CBG-C4** | 0.002 | 0.000 | | 0.002 | | 0.000 | 0.000 |
| **CBGM** | 0.001 | 0.000 | | 0.002 | | 0.000 | 0.000 |
| **CBGMA** | 0.000 | 0.000 | | 0.000 | | 0.000 | 0.000 |
| **CBGO** | 0.000 | 0.000 | | 0.000 | | 0.000 | 0.000 |
| **CBGOA** | 0.000 | 0.001 | | 0.000 | | 0.000 | 0.000 |
| **CBGV** | 0.008 | 0.000 | | 0.013 | | 0.000 | 0.000 |
| **CBGVA** | 0.000 | 0.000 | | 0.000 | | 0.000 | 0.000 |
| **CBL** | 0.026 | 0.000 | | 0.000 | | 0.327 | 0.000 |
| **CBN** | 0.118 | 0.013 | | 0.002 | | 1.479 | 0.002 |
| **CBNA** | 0.000 | 0.000 | | 0.000 | | 0.000 | 0.000 |
| **CBNA-C4** | 0.000 | 0.000 | | 0.000 | | 0.000 | 0.000 |
| **CBN-C4** | 0.000 | 0.000 | | 0.001 | | 0.000 | 0.000 |
| **CBND** | 0.008 | 0.000 | | 0.015 | | 0.000 | 0.000 |
| **CBNDA** | 0.001 | 0.000 | | 0.001 | | 0.000 | 0.000 |
| **CBNDVA** | 0.000 | 0.001 | | 0.000 | | 0.000 | 0.000 |
| **CBNM** | 0.000 | 0.000 | | 0.000 | | 0.000 | 0.000 |
| **CBNMA** | 0.000 | 0.000 | | 0.000 | | 0.000 | 0.000 |
| **CBNO** | 0.000 | 0.000 | | 0.000 | | 0.000 | 0.000 |
| **CBNOA** | 0.000 | 0.000 | | 0.000 | | 0.000 | 0.000 |
| **CBNV** | 0.005 | 0.000 | | 0.009 | | 0.000 | 0.000 |
| **CBNVA** | 0.000 | 0.000 | | 0.000 | | 0.000 | 0.000 |
| **CBT-1** | 0.065 | 0.016 | | 0.099 | | 0.086 | 0.008 |
| **CBT-2** | 0.015 | 0.004 | | 0.015 | | 0.028 | 0.001 |
| **CBT-3** | 0.047 | 0.023 | | 0.067 | | 0.128 | 0.009 |
| **CBTA-1** | 0.000 | 0.001 | | 0.000 | | 0.000 | 0.000 |
| **CBTA-3** | 0.000 | 0.000 | | 0.002 | | 0.001 | 0.000 |
| **CBTV-1** | 0.004 | 0.139 | | 0.000 | | 0.000 | 0.000 |
| **CBTV-3** | 0.000 | 0.013 | | 0.002 | | 0.000 | 0.000 |
| **d8-THC** | 0.014 | 0.000 | | 0.000 | | 0.159 | 0.000 |
| **d9-THC** | 1.620 | 0.000 | | 0.000 | | 19.001 | 0.000 |
| **d9-THCA** | 0.000 | 0.000 | | 0.000 | | 0.000 | 0.000 |
| **d9-THCA-C4** | 0.000 | 0.000 | | 0.000 | | 0.000 | 0.000 |
| **d9-THC-C4** | 0.000 | 0.000 | | 0.000 | | 0.084 | 0.000 |
| **d9-THCM** | 0.000 | 0.000 | | 0.000 | | 0.000 | 0.000 |
| **d9-THCMA** | 0.000 | 0.000 | | 0.000 | | 0.000 | 0.000 |
| **d9-THCO** | 0.000 | 0.000 | | 0.000 | | 0.000 | 0.000 |
| **d9-THCOA** | 0.000 | 0.000 | | 0.000 | | 0.000 | 0.000 |
| **d9-THCV** | 0.150 | 0.000 | | 0.245 | | 0.000 | 0.000 |
| **d9-THCVA** | 0.000 | 0.000 | | 0.000 | | 0.000 | 0.000 |
| **OH-CBN** | 0.003 | 0.000 | | 0.004 | | 0.015 | 0.000 |
| **OH-CBNA** | 0.000 | 0.000 | | 0.000 | | 0.000 | 0.000 |
| **SesquiCBG** | 0.025 | 0.000 | | 0.000 | | 0.228 | 0.000 |
| **SesquiCBGA** | 0.000 | 0.000 | | 0.000 | | 0.000 | 0.000 |
| **313-16b** | 0.273 | 0.000 | | 0.000 | | 2.981 | 0.000 |
| **327-13a** | 0.201 | 0.006 | | 0.330 | | 0.015 | 0.000 |
| **327-13b** | 0.178 | 0.012 | | 0.270 | | 0.023 | 0.000 |
| **327-13c** | 0.249 | 0.064 | | 0.174 | | 0.830 | 0.047 |
| **329-11a** | 0.011 | 0.592 | | 0.001 | | 0.000 | 0.000 |
| **329-11b** | 0.118 | 0.002 | | 0.194 | | 0.000 | 0.000 |
| **329-11c** | 0.016 | 0.007 | | 0.020 | | 0.170 | 0.000 |
| **329-11d** | 0.000 | 0.000 | | 0.000 | | 0.002 | 0.000 |
| **331-18a** | 2.042 | 0.003 | | 3.308 | | 0.003 | 0.000 |
| **331-18b** | 0.543 | 0.000 | | 0.511 | | 0.007 | 0.037 |
| **331-18c** | 0.083 | 0.130 | | 0.126 | | 0.029 | 0.098 |
| **331-18d** | 0.073 | 0.000 | | 0.010 | | 0.436 | 0.000 |
| **357-16a** | 0.000 | 0.000 | | 0.000 | | 0.000 | 0.000 |
| **361-17a** | 0.003 | 0.019 | | 0.005 | | 0.002 | 0.000 |
| **361-17b** | 0.003 | 0.125 | | 0.000 | | 0.002 | 0.000 |
| **371-14a** | 0.002 | 0.002 | | 0.003 | | 0.001 | 0.001 |
| **371-14b** | 0.000 | 0.000 | | 0.000 | | 0.000 | 0.000 |
| **373-12a** | 0.000 | 0.004 | | 0.000 | | 0.000 | 0.000 |
| **373-12b** | 0.000 | 0.000 | | 0.000 | | 0.000 | 0.001 |
| **373-12c** | 0.000 | 0.000 | | 0.000 | | 0.000 | 0.000 |
| **373-12d** | 0.000 | 0.000 | | 0.000 | | 0.000 | 0.000 |
| **373-15b** | 0.000 | 0.002 | | 0.000 | | 0.003 | 0.000 |
| **373-15c** | 0.383 | 0.013 | | 0.002 | | 0.021 | 4.478 |
| **375-19a** | 0.003 | 0.016 | | 0.004 | | 0.002 | 0.001 |
| **375-19b** | 0.000 | 0.005 | | 0.000 | | 0.000 | 0.000 |
| **375-19c** | 0.000 | 0.000 | | 0.000 | | 0.000 | 0.000 |
| **417-15a** | 0.000 | 0.001 | | 0.000 | | 0.000 | 0.000 |
