# Supplementary material for: Cannabinoid combination targets NOTCH1-mutated T-cell acute lymphoblastic leukemia through the integrated stress response pathway
Source: eLife. 2024 Sep 11;12:RP90854. doi: 10.7554/eLife.90854 (PMC11390110; doi:10.7554/eLife.90854)
Supplement: Supplementary file 2. [file elife-90854-supp2.docx]

Supplementary file 2. Phytocannabinoid concentrations by UHPLC/LC-MS of Cannabis fraction 2 peaks

|  | **P1** | **P2** | **P3** | **P4** | **P5** |
| --- | --- | --- | --- | --- | --- |
| **331-18a** | 181.0502 | 5.0040 | 0.1243 | 0.1195 | 0.0000 |
| **CBD** | 4.0371 | 0.0915 | 1.0801 | 1.0917 | 109.3740 |
| **CBDVA** | 1.1604 | 0.0411 | 0.0000 | 0.0000 | 0.0000 |
| **CBDA** | 0.9107 | 0.0000 | 0.0385 | 11.7560 | 0.0000 |
| **CBND** | 0.6878 | 0.0307 | 0.0000 | 0.0000 | 0.0000 |
| **THCA** | 0.5969 | 0.0000 | 0.1629 | 0.1648 | 0.0000 |
| **329-11b** | 0.3289 | 0.2398 | 0.0089 | 0.0151 | 0.0000 |
| **CBT-2** | 0.1802 | 0.0135 | 0.0152 | 0.0147 | 0.0000 |
| **373-15c** | 0.1545 | 0.0876 | 0.0558 | 0.0565 | 0.0000 |
| **327-13c** | 0.0546 | 0.0000 | 0.0192 | 0.0493 | 0.1371 |
| **CBCA** | 0.0401 | 0.0000 | 0.0000 | 0.0000 | 0.0000 |
| **CBDV** | 0.0246 | 93.2161 | 0.8774 | 0.9112 | 0.0000 |
| **327-13b** | 0.0212 | 0.0000 | 0.1556 | 0.1526 | 0.0000 |
| **CBN** | 0.0152 | 0.0000 | 0.0989 | 0.0870 | 0.0000 |
| **327-13a** | 0.0000 | 0.9210 | 0.1657 | 0.1922 | 0.0000 |
| **CBGV** | 0.0000 | 0.2160 | 0.0000 | 0.0000 | 0.0000 |
| **CBD-C4** | 0.0000 | 0.0000 | 20.5724 | 0.1509 | 0.0000 |
| **CBT-3** | 0.0000 | 0.0000 | 0.0783 | 0.0844 | 0.0000 |
| **361-17a** | 0.0000 | 0.0000 | 0.0438 | 0.0416 | 0.0000 |
| **CBG-C4** | 0.0000 | 0.0000 | 0.0254 | 0.0262 | 0.0000 |
| **373-15b** | 0.0000 | 0.0000 | 0.0244 | 0.0231 | 0.0000 |
| **329-11d** | 0.0000 | 0.0000 | 0.0179 | 0.0198 | 0.0000 |
| **CBEA** | 0.0000 | 0.0000 | 0.0135 | 0.0361 | 0.0000 |
| **CBGA-C4** | 0.0000 | 0.0000 | 0.0079 | 0.0079 | 0.0000 |
| **CBDA-C4** | 0.0000 | 0.0000 | 0.0030 | 0.0030 | 0.0000 |
| **331-18b** | 0.0000 | 0.0000 | 0.0000 | 2.0747 | 0.3356 |
| **CBNV** | 0.0000 | 0.0000 | 0.0000 | 0.0338 | 0.0000 |
| **CBG** | 0.0000 | 0.0000 | 0.0000 | 0.0000 | 0.4119 |
| **CBE** | 0.0000 | 0.0000 | 0.0000 | 0.0000 | 0.0142 |
