# Supplementary material for: Cannabinoid combination targets NOTCH1-mutated T-cell acute lymphoblastic leukemia through the integrated stress response pathway
Source: eLife. 2024 Sep 11;12:RP90854. doi: 10.7554/eLife.90854 (PMC11390110; doi:10.7554/eLife.90854)
Supplement: Supplementary file 3. [file elife-90854-supp3.docx]

**Supplementary file 3: ^1^H and ^13^C peak assignments and chemical shifts of CBD and 331-18A**

|  | **CBD^a^** | | **331-18A** | |
| --- | --- | --- | --- | --- |
|  | **^1^H** | **^13^C** | **^1^H** | **^13^C** |
| **1** | 3.85 | 37.08 | 3.83 | 32.63 |
| **2** | 5.57 | 123.96 | 5.71 | 123.45 |
| **3** | - | 140.21 | - | 140.20 |
| **4** | 2.10, 2.23 | 30.31 | 2.06, 2.13 | 27.08 |
| **5** | 1.77, 1.82 | 28.28 | 1.73, 1.97 | 22.66 |
| **6** | 2.39 | 46.10 | 1.90 | 48.24 |
| **7** | 1.79 | 23.75 | 1.81 | 23.82 |
| **8** | - | 149.38 | - | 75.19 |
| **9** | 1.66 | 20.44 | 1.25 | 25.97 |
| **10** | 4.55, 4.67 | 110.86 | 1.26 | 29.70 |
| **1'** | - | 113.63 | - | 114.66 |
| **2'** | - | 155.95 | - | 155.97 |
| **2'-OH** | 6.05 | - | 6.61 | - |
| **3'** | 6.29 | 109.68 | 6.26 | 109.44 |
| **4'** | - | 143.02 | - | 143.52 |
| **5'** | 6.16 | 107.88 | 6.33 | 109.44 |
| **5'-OH** | 4.77 | - | 7.64 | - |
| **6'** | - | 153.78 | - | 154.23 |
| **1''** | 2.43 | 35.44 | 2.45 | 35.49 |
| **2''** | 1.55 | 30.69 | 1.57 | 30.74 |
| **3''** | 1.27 | 31.46 | 1.29 | 31.52 |
| **4''** | 1.30 | 22.55 | 1.31 | 22.56 |
| **5''** | 0.87 | 14.09 | 0.88 | 14.08 |

^a^ Peak assignments are in close agreement with the literature (*33*).
