# Supplementary material for: Cannabinoid combination targets NOTCH1-mutated T-cell acute lymphoblastic leukemia through the integrated stress response pathway
Source: eLife. 2024 Sep 11;12:RP90854. doi: 10.7554/eLife.90854 (PMC11390110; doi:10.7554/eLife.90854)
Supplement: Supplementary file 4. [file elife-90854-supp4.docx]

Supplementary file 4. Ten most increased- and decreased-abundance genes following treatment of MOLT-4 cells with the whole extract according to Affymetrix

|  | **Increased abundance ^a^** | | **Decreased abundance ^a^** | |
| --- | --- | --- | --- | --- |
| **1** | *SLC7A11* | 34.2 | *EYA4* | -7.25 |
| **2** | *CHAC1* | 27.48 | *CHTF8* | -6.83 |
| **3** | *JUN* | 25.75 | *CD180* | -5.74 |
| **4** | *ID2* | 21.51 | *DHCR7* | -5.51 |
| **5** | *MT1L* | 19.88 | *FSIP1* | -5.28 |
| **6** | *SNAI1* | 17.41 | *DEFB113* | -5.25 |
| **7** | *SLC43A1* | 16.96 | *C15orf65* | -5.22 |
| **8** | *MT1X* | 16.57 | *KRTAP10-10* | -5.2 |
| **9** | *EGR1* | 16.03 | *GRIA2* | -5.11 |
| **10** | *CITED2* | 15.98 | *OR5T3* | -5.02 |

^a^ Results are presented as gene expression fold change compared to vehicle treatment. The full list of genes that are up-regulated or down-regulated upon treatment with Extract 12 is available in the Gene Expression Omnibus (GEO) repository, GSE154287, <https://www.ncbi.nlm.nih.gov/geo/query/acc.cgi?acc=GSE154287>.
